# Parallel Chemical Genetic and Genome-Wide RNAi Screens Identify Cytokinesis Inhibitors and Targets

**DOI:** 10.1371/journal.pbio.0020379

**Published:** 2004-10-05

**Authors:** Ulrike S Eggert, Amy A Kiger, Constance Richter, Zachary E Perlman, Norbert Perrimon, Timothy J Mitchison, Christine M Field

**Affiliations:** **1**Department of Systems Biology, Harvard Medical SchoolBoston, MassachusettsUnited States of America; **2**Institute of Chemistry and Cell Biology, Harvard Medical SchoolBoston, MassachusettsUnited States of America; **3**Department of Genetics, Harvard Medical SchoolBoston, MassachusettsUnited States of America; **4**Howard Hughes Medical Institute, Harvard Medical SchoolBoston, MassachusettsUnited States of America

## Abstract

Cytokinesis involves temporally and spatially coordinated action of the cell cycle and cytoskeletal and membrane systems to achieve separation of daughter cells. To dissect cytokinesis mechanisms it would be useful to have a complete catalog of the proteins involved, and small molecule tools for specifically inhibiting them with tight temporal control. Finding active small molecules by cell-based screening entails the difficult step of identifying their targets. We performed parallel chemical genetic and genome-wide RNA interference screens in *Drosophila* cells, identifying 50 small molecule inhibitors of cytokinesis and 214 genes important for cytokinesis, including a new protein in the Aurora B pathway (Borr). By comparing small molecule and RNAi phenotypes, we identified a small molecule that inhibits the Aurora B kinase pathway. Our protein list provides a starting point for systematic dissection of cytokinesis, a direction that will be greatly facilitated by also having diverse small molecule inhibitors, which we have identified. Dissection of the Aurora B pathway, where we found a new gene and a specific small molecule inhibitor, should benefit particularly. Our study shows that parallel RNA interference and small molecule screening is a generally useful approach to identifying active small molecules and their target pathways.

## Abstract 2

Parallel genetic and chemical screens are used to find small molecules that interfere with cell division and to identify the specific targets for these molecules

## Introduction

Small molecule inhibitors are useful tools for studying dynamic biological processes. Compared to mutations and RNA interference (RNAi), cell-permeable small molecules allow inhibition of protein function with precise temporal control, and may also spur development of new therapeutics. One approach to finding useful small molecules is phenotypic screening, in which cells are treated with small molecules from a library and scored for inhibition of the process of interest. The rate-limiting step in this approach is identifying the cellular targets of active small molecules. Traditionally, the targets of small molecules have been identified by methods based on physical affinity, for example, affinity chromatography (Harding et al. 1989). These require chemical modification of the small molecule and suffer the limitation that irrelevant proteins will bind in addition to the authentic target. A complementary method is to use information on the biological activity of the small molecule to identify the cellular pathway it perturbs. In some cases an educated guess can be successful (Mayer et al. 1999), but to be generally useful, the biological activity of a small molecule would need to be systematically compared to the effect of perturbing different cellular pathways. Currently, the most general method for systematically perturbing pathways and collecting phenotypic information is RNAi, which can be used to inhibit protein function on a genome-wide basis. Here, we develop a parallel screening strategy for finding small molecules that inhibit the biological process of cytokinesis, the genes required for this process, and, by cross-comparison of phenotypes, information on the protein targets of the small molecules.

Cytokinesis is the final step of cell division, when the daughter cells are physically separated by constriction of a cleavage furrow. Complex spatiotemporal coordination of several cell systems, including the microtubule and actin cytoskeletons, the cell cycle engine, and vesicle trafficking, is required for furrow positioning, assembly, ingression, and eventual cell separation. Some important components of the cleavage furrow, for example, actin (Schroeder 1973), Myosin (Mabuchi and Okuno 1977), and Anillin (Oegema et al. 2000), have been identified, as well as signaling systems that position and regulate the furrow, such as Aurora B (Carmena and Earnshaw 2003) and Polo (Carmena et al. 1998) kinases and Rho family GTPases (Prokopenko et al. 2000). Since many of these proteins also play roles in additional cellular processes, analysis of their function in cytokinesis by genetic methods can be difficult, and small molecule tools would be useful. To date, only three cytokinesis proteins, actin, Myosin II, and Aurora B kinase, have been targeted with small molecules, but even this limited set has been very useful. For example, the actin inhibitor cytochalasin was used to discover the central role of actin in cytokinesis (reviewed in Peterson and Mitchison 2002), and the Myosin II inhibitor, blebbistatin, provided insight into the coordination of different processes in cytokinesis (Straight et al. 2003). Small molecule inhibitors of Aurora B kinase have recently been reported, but their effect on cytokinesis has yet to be investigated in detail (Ditchfield et al. 2003; Hauf et al. 2003).

Although several key cytokinesis proteins are known, we lack a complete list of proteins required for cytokinesis in any organism. In a genome-wide study, 98 proteins were reported to localize to the bud neck, the site of cytokinesis in Saccharomyces cerevisiae (Huh et al. 2003), but their functional roles have not yet been systematically investigated. A proteomic screen that identified many components of mammalian midbodies, organelle-like remnants of the cleavage furrow, was reported recently (Skop et al. 2004) and several small-scale RNAi studies have been conducted in *Drosophila* (Somma et al. 2002; Goshima and Vale 2003; Kiger et al. 2003; Rogers et al. 2003). These screens identified genes required for cytokinesis, but did not assay an entire genome. Genome-wide RNAi screens have been carried out in Caenorhabditis elegans and in *Drosophila* cells, but they did not focus on genes required for cytokinesis (Kamath et al. 2003; Boutros et al. 2004).

## Results/Discussion

### Parallel Screening Protocols

To identify all genes required for cytokinesis, and small molecules that target their products, we developed an assay for both comprehensive functional genomic and large-scale chemical genetic screens in cultured *Drosophila* cells. We chose this system because of both the availability of genome-wide RNAi resources (Boutros et al. 2004) and the ease of RNAi in *Drosophila* cells. *Drosophila* cells can take up long pieces of double-stranded RNA (dsRNA) from the culture medium and process them into small interfering RNAs without triggering an interferon response, in contrast to mammalian cells (Clemens et al. 2000; Elbashir et al. 2001). Furthermore, use of a single targeting dsRNA is efficient for the knockdown of a specific gene in each experiment. Therefore, we were able to screen a library of existing dsRNAs with an average length of 408 bp (Hild et al. 2003) to functionally test nearly all *Drosophila* genes for roles in cytokinesis.

Cells that undergo mitosis normally, but fail cytokinesis, acquire two nuclei. This phenotype is a specific and irreversible consequence of cytokinesis failure, and can be scored by automated fluorescence microscopy. *Drosophila* Kc_167_ cells were cultured together with either gene-specific dsRNAs or discrete small molecules in optical-bottom 384-well plates. In total, we screened 19,470 dsRNAs covering more than 90% of the annotated genome in triplicate at the Drosophila RNAi Screening Center (http://www.flyrnai.org) and over 51,000 small molecules at the Institute of Chemistry and Cell Biology (http://iccb.med.harvard.edu). The cells were incubated for 4 d in the presence of dsRNAs to allow for depletion and turnover of targeted gene products, or 2 d for small molecules to permit each cell to complete at least one cell cycle. After fixation, cells were stained with amine-reactive tetramethylrhodamine-NHS ester to visualize total cytoplasm and Hoechst dye to visualize DNA. In the RNAi screen, microtubules were visualized by immunofluorescence. Cells were imaged by automated fluorescence microscopy, and assay wells containing a high frequency of binucleate cells were identified by a combination of automated image analysis and visual inspection.

### Small Molecule Screening Results

From approximately 51,000 small molecules that included a mixture of commercial “drug-like” molecules, natural product extracts, and natural-product-like libraries synthesized at the Institute of Chemistry and Cell Biology (http://iccb.med.harvard.edu, we identified 50 small molecule inhibitors of cytokinesis, and selected 25 of the most potent and readily available for further analysis (Table S1). This structurally diverse group, which we named binucleines 1–25, included 12 small molecules from commercial libraries, ten known bioactives, and three natural product extracts. We screened at a nominal concentration of 12.5 μg/ml (approximately 25 μM) and retested the effect of our 25 small molecule inhibitors on *Drosophila* tissue culture cells at three different concentrations: 100 μM, 30 μM, and 10 μM (Table S1). To determine cross-reactivity with other species, we also assayed cytokinesis inhibition in HeLa (64%, 16/25 active) and BSC-1 (52%, 13/25 active) tissue culture cells as well as growth inhibition in drug-sensitive S. cerevisiae (48%, 12/25 active) (see Table S1).

Since most compounds currently known to inhibit cytokinesis are natural product actin binders, we tested if the small molecule inhibitors affected actin polymerization. Binucleines 4, 6, 24, and 25 inhibited pyrene–actin polymerization in a pure protein assay (data not shown). Binucleines 24 and 25 are the actin binders cytochalasin D and jasplakinolide, which were included in our small molecule collection as control compounds. Binucleine 4 is a natural product extract from *Ircinia ramosa,* which contains swinholide A, a known actin binder, as its active ingredient (F. C. Schroeder and J. Clardy, personal communication). To learn more about the cellular targets of the remaining compounds, we proceeded with our plan to systematically compare small molecule and RNAi phenotypes.

### Genome-Wide RNAi Screening Results

**Figure 1:**
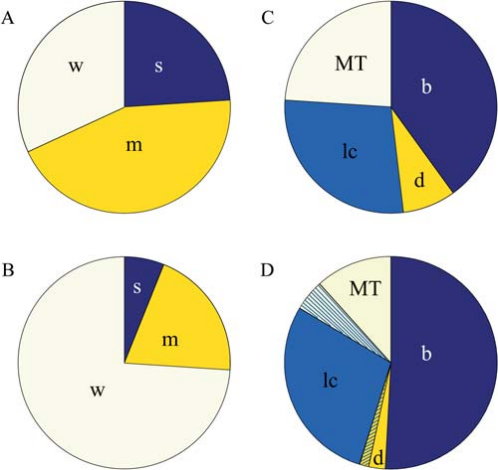
Distribution of Active Small Molecules and Genes Targeted by RNAi Identified by Penetrance of Binucleate Phenotype and by Phenotypic Classes (A and B) Penetrance of binucleate phenotype for small molecules (A) and RNAi hits (B). (A) For the small molecules, 24% (6/25) were strong (s), 44% (11/25) medium (m), and 32% (8/25) weak (w). (B) For the RNAi hits 6% (13/2114) were strong (s), 20% (43/214) medium (m), and 74% (158/214) weak (w). In a weakly penetrant phenotype, the binucleate level was increased by more than 1.25-fold relative to the two neighboring wells in at least two experiments. In a medium penetrance phenotype, the binucleate level was above 4%, or four times as high as the neighboring wells. In a strongly penetrant phenotype, the binucleate level was above 15%. The average binucleate level in controls was approximately 1%. (C and D) Phenotypic classes for small molecules (C) and genes targeted by dsRNAs (D). (C) For the small molecules, 40% (10/25) were binucleate (b; Figure 2A), 8% (2/25) binucleate with large, diffuse DNA (d; Figure 2B), 28% (7/25) binucleate with low cell count (lc; Figure 2C), and 24% (6/25) binucleate with microtubule extensions (MT; Figure 2D). (D) For the RNAi hits, 51% (109/214) were binucleate (b; Figure 2A), 2% (5/214) binucleate with large, diffuse DNA (d; Figure 2B), 29% (62/214) binucleate with low cell count (lc; Figure 2C), and 12% (25/214) binucleate with microtubule extensions (MT; Figure 2D). In addition, 5% (10/214) were binucleate with low cell count and microtubule extensions, and 1% (3/214) were binucleate with low cell count and large, diffuse DNA.

**Figure 2:**
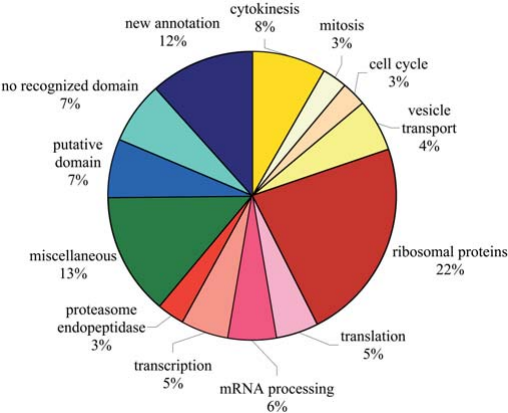
Predicted Functional Annotations of 214 Genes Associated with RNAi Binucleate Phenotypes Functional groups were assigned using Gene Ontology information presented in FlyBase or the literature (see Table S4). Genes involved in processes associated with cytokinesis are shown in shades of yellow, nucleic acid and protein synthesis and degradation in shades of red, and uncharacterized genes in shades of blue. The uncharacterized genes encode protein sequences that predict recognizable domains (“putative domain”), no recognizable domains (“no recognized domain”), or new gene predictions from the reannotation of the *Drosophila* genome used as the basis of the dsRNA library (“new annotation”).

We identified dsRNAs corresponding to 214 genes with phenotypes important for cytokinesis (Table S2). Only dsRNAs that resulted in a binucleate phenotype in at least two of the three replicate screens were summarized in our final results (Table S3). These genes resulted in either a strong, medium, or weak increase in frequency of binucleate cells (Figure 1) and represented a diverse range of predicted cellular functions (Figure 2; Table S4), reflecting the complexity of cytokinesis. Of the RNAi phenotypes, 20% identified genes previously directly implicated (Table S5 and references therein) or involved in processes associated with cytokinesis. Eleven of the strong phenotypes identified such genes, including two copies of actin *(Act57B* and *Act5C),* Myosin heavy chain *(zipper),* Anillin *(scraps),* a formin *(diaphanous),* Rho GTPase *(Rho1)* and its known guanine nucleotide exchange factor *(pebble)* and GTPase-activating protein *(RacGAP50C),* a kinesin *(pavarotti),* Citron kinase *(CG10522),* Aurora B kinase *(ial),* and a PRC1 homolog *(fascetto)*. We discovered one new gene essential for cytokinesis (*CG4454,* see discussion below), increasing the number of specific essential proteins confirmed by RNAi to thirteen (the twelve genes listed above and *INCENP;* see Table S5). Although required for cytokinesis (Adams et al. 2001) and successfully resynthesized for later experiments, *INCENP* was not identified in our screen because of failure in *INCENP* dsRNA synthesis.

Cytokinesis is a complex, multistep process, unlikely to be regulated and executed by only thirteen proteins, suggesting that many other proteins with less stringent requirements are also involved. Our screen identified 201 genes with a loss-of-function phenotype of a medium or weakly penetrant cytokinesis failure. Of these genes, 54 were predicted to encode proteins with unknown function, including 25 that were targeted with dsRNA on the basis of new gene model predictions (Hild et al. 2003). The remaining genes had a variety of predicted functions, including cell cycle regulation and vesicle transport. Cytokinesis is known to require insertion of new plasma membrane (Finger and White 2002), consistent with our identification of genes involved in vesicle transport (12 genes). We were surprised, however, that this group included most of the components of the coatomer complex COPI (5/7 COPI subunits). The COPI complex is thought to be involved in retrograde transport from Golgi apparatus to endoplasmic reticulum, and its role in cytokinesis remains to be elucidated. An unexpected functional group, identified mostly with weak phenotypes, included genes involved in nucleic acid and protein synthesis and degradation, including a large number of ribosomal proteins. We subjected nine of these genes to further analysis. In seven of nine cases, filamentous actin staining was very weak, while other proteins such as tubulin and Myosin were of normal abundance, suggesting that the phenotype may result from low-level synthesis of actin or other cortical components (data not shown). Since a library of a single long dsRNA per gene was used in this screen, it is conceivable that some phenotypes are due to off-target effects. The approximately 400-nt dsRNAs are processed into smaller small interfering RNAs, and if appropriately processed, could cross-hybridize partially or completely with identical sequences in mRNA corresponding to other genes (Bartel 2004; Tijsterman and Plasterk 2004). Of 214 dsRNAs identified in our screen, 57 had a potential 21-nt overlap with other genes (see Table S3). In the majority of these cases (39/57), full-length dsRNA corresponding to the potential cross-match gene did not itself score. Some related genes, for example, the five copies of actin identified in our screen, show high homology and are therefore expected to contain overlapping dsRNA sequences.

### Comparison of RNAi Screen to Other Screens

Since one of our goals was to create an inventory of all genes required for cytokinesis, it is important to evaluate the success rate of the genome-wide RNAi screen with respect to other published screens and to the cytokinesis literature in general. Four small-scale screens have examined the role of specific genes in cytokinesis in *Drosophila* cells (Somma et al. 2002; Goshima and Vale 2003; Kiger et al. 2003; Rogers et al. 2003). Results from our genome-wide screen correlate well with data from the four smaller screens and other experiments, indicating that the field is converging on a consensus of genes absolutely required for cytokinesis (see Table S5). Ten genes, reported elsewhere with RNAi binucleate phenotypes in *Drosophila* cells, did not score in our screen (*INCENP* [Adams et al. 2001]; *syx1A* [Somma et al. 2002]; *profilin*, *aip1 [CG10724],* and *capt* [Rogers et al. 2003]; and *kst, Toll, Toll-4, bazooka,* and *kekkon* [Kiger et al. 2003]). The dsRNA targeting these genes, apart from *INCENP,* passed quality control, suggesting alternative explanations for the differences between various RNAi experiments. Three of the smaller screens were carried out in *Drosophila* S2 cells (Somma et al. 2002; Goshima and Vale 2003; Rogers et al. 2003), which may differentially express or require certain proteins. The timing of RNAi experiments may also contribute to differences that were observed. We exposed cells to dsRNAs for 4 d, balancing sufficient depletion with potentially detrimental effects of prolonged culture and exposure of cells to dsRNA. With an average cell cycle of 24 h, 4 d may be too short to completely deplete very stable proteins. For example, depletion of the Myosin II regulatory light chain *(spaghetti squash)* resulted in a weak phenotype, whereas depletion of its complex partner encoded by *zipper,* the Myosin II heavy chain, resulted in a much higher frequency of binucleates. This illustrates the importance of identifying medium and weak phenotypes. Genes with weaker binucleate phenotypes may also be significant because depletion of cytokinesis proteins with multiple functions during the cell cycle can cause arrest prior to cytokinesis, diminishing the likelihood of detecting the phenotype in unsynchronized cells. Overlap between our screen and a recently published proteomic analysis of the midbody (Skop et al. 2004) highlights the importance of identifying genes with weaker binucleate phenotypes. For example, the Arp2/3 complex was not thought to play a role in cytokinesis in metazoans, but components of this complex were identified in both approaches. Eventually, a combination of different methods will result in a definitive list of all proteins involved in cytokinesis.

### Systematic Comparisons between RNAi and Small Molecule Phenotypes

**Figure 3:**
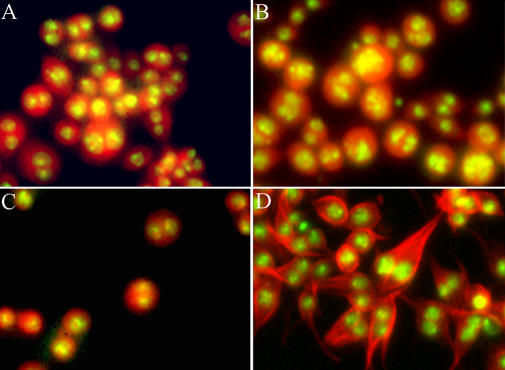
Phenotypic Classes The phenotypic classes are (A) binucleate (*CG10522* RNAi) and binucleate with (B) large, diffuse DNA (*aurora B* RNAi), (C) low cell count (*RpS18* RNAi), or (D) microtubule extensions (*Act5C* RNAi). In (A), (B), and (C), the cytoplasm (tetramethylrhodamine stain) of Kc_167_ cells is shown in red and DNA in green. In (D), tubulin is shown in red and DNA in green. See Table S2 for full classification.

**Figure 4:**
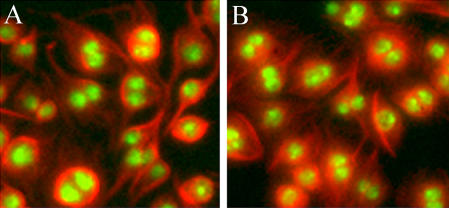
Kc_167_ Cells Exposed to dsRNA Targeting *Act5C* or to Cytochalasin D The cells were exposed to dsRNA targeting *Act5C* for 4 d (A) or to cytochalasin D at 5 μM for 48 h (B). Tubulin is shown in red, DNA in green.

Following our strategy to systematically compare chemical genetic and functional genomic data, we classified the data from both screens into four phenotypic groups (summarized in Figure 1C and 1D): (1) binucleate cell phenotype only (Figure 3A), or a combination of binucleate cells with an additional phenotype of (2) large, diffuse DNA (Figure 3B), (3) low total cell count (Figure 3C), or (4) microtubule extensions (Figure 3D). The initial classification into four phenotypic groups was based on the raw screening data, where our parameters were whole cell, DNA, or tubulin staining. While these phenotypic classes are useful for global analysis and preliminary characterization, there were too many genes in each group to allow meaningful comparisons between small molecule and RNAi phenotypes. Therefore, we selected 40 genes and 25 small molecules for more detailed analysis. To ascertain specific defects in cytokinesis, we determined by immunolocalization the behavior of 15 proteins involved in cytokinesis. Our bank of reagents included antibodies to proteins that are normally found in the cleavage furrow such as actin (phalloidin), Anillin, Myosin II, and the septin protein Peanut; proteins involved in the regulation of cytokinesis such as Aurora B (Giet and Glover 2001), RhoA, Pebble (Prokopenko et al. 1999), and Polo kinase (Tavares et al. 1996); proteins involved in other aspects of cytokinesis like Diaphanous, Lava-lamp (Sisson et al. 2000), and Pavarotti; and proteins that report on the stage of cytokinesis or the state of the cell cycle such as Lamin (Risau et al. 1981), phospho-Histone H3, and tubulin. As a specific example of this detailed analysis, the phenotypes for Aurora B kinase and CG4454 are discussed below.

### Small Molecules Can Result in Additional Phenotypes

We identified phenotypes common to both datasets, but the detailed phenotypic analyses did not match exactly, with more phenotypic subclasses distinguished with the small molecules. Two considerations may account for the existence of additional phenotypic categories for small molecules. One is timing. During our detailed secondary analysis, we added small molecules to cells for variable amounts of time (3 h to 48 h). When cells were exposed to a drug for a short time, we were able to analyze localization of furrow components immediately after cytokinesis failure. These phenotypes became less apparent upon longer exposure because the long delay gave cells the opportunity to disassemble residual furrow structures. When cells were exposed to dsRNAs for days, long, variable delays between cytokinesis failure and fixation may have obscured interesting phenotypes, which could be revealed by subsequent real-time imaging experiments (Goshima and Vale 2003). The other consideration is potential gain-of-function effects of small molecules. For example, a natural product extract from Cowania mexicana containing a cucurbitacin (M. Fujita and J. Clardy, personal communication) caused clusters of filamentous actin to accumulate in interphase cells, in addition to completely blocking cytokinesis. Since no dsRNA caused this phenotype, we suspect it is a gain-of-function effect of the small molecule, whose mechanism we will pursue.

### Two Sub-Phenotypes Correlate in Both Small Molecule and RNAi Datasets

Systematic comparison between the phenotypic categories based on detailed immunofluorescence analysis of both screens did, however, allow us to connect small molecules to two specific pathways involved in cytokinesis, namely actin cortex integrity and the Aurora B pathway. Both dsRNAs and small molecules that weakened the actin cortex caused microtubule-rich extensions to protrude from interphase cells as well as failure of cytokinesis (Figure 4). These included dsRNAs targeted against several actin genes (see Table S2) and three natural product small molecules known to target actin that were present in our small molecule collection (cytochalasin D, jasplakinolide, and swinholide A [from Ircinia ramosa extract]). This sub-phenotype represents a portion of the genes identified as “binucleate with microtubule extensions” shown in Figure 3D.

**Figure 5:**
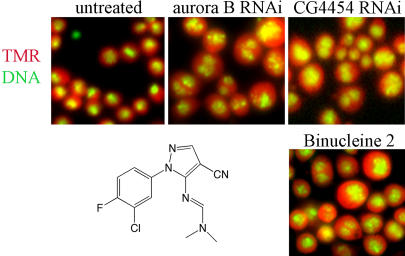
Kc_167_ Cells Untreated or Exposed to *aurora B* dsRNA, *borr (CG4454)* dsRNA, or Binucleine 2 TMR-stained cells were untreated, or treated with dsRNA for 4 d or binucleine 2 (50 μM) for 2 d. TMR is shown in red, DNA in green. The chemical structure of binucleine 2 is also shown.

A second phenotypic class exhibited a high incidence of both mitosis and cytokinesis defects, a sub-phenotype of the category “binucleate with diffuse DNA” (see Figure 3B). Mitosis was abnormal, with malformed spindles and misaligned chromosomes, resulting in large, diffuse arrangements of DNA in binucleate cells (Figure 5). Individual depletion of any of three proteins encoded by *aurora B*, *INCENP,* and *CG4454*, or addition of one small molecule *N′*-[1-(3-chloro-4-fluorophenyl)-4-cyano-1H-pyrazol-5-yl]-*N*,*N*-dimethyliminoformamide (binucleine 2; Figure 5), caused this phenotype.

### CG4454 RNAi Phenotype and Localization Matches Chromosomal Passenger Proteins

**Figure 6:**
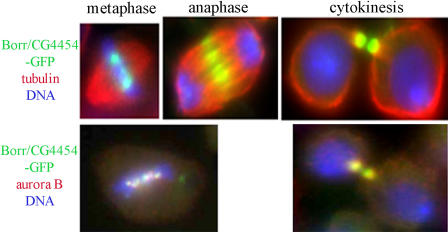
Kc_167_ Cells Transfected with Borr-GFP In the top row, cells in metaphase, anaphase, and cytokinesis are shown. Borr-GFP is shown in green, tubulin in red, and DNA in blue. The bottom row shows cells in metaphase and cytokinesis. Borr-GFP is shown in green, Aurora B in red, and DNA in blue.

**Figure 7:**
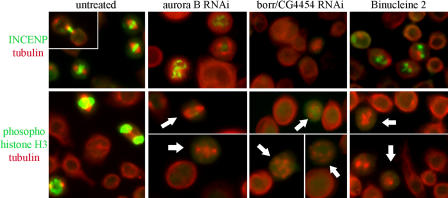
Kc_167_ Cells Untreated or Exposed to *aurora B* dsRNA, *borr (CG4454)* dsRNA, or Binucleine 2 INCENP-stained cells in the top row were untreated or treated with *aurora B* dsRNA for 5 d, *borr (CG4454)* dsRNA for 3 d, or binucleine 2 (20 μM) for 4 h. Phospho-Histone H3 stained cells in the bottom row were untreated or treated with dsRNA for 4 d or binucleine 2 (20 μM) for 4 h. White arrows indicate absence of phospho-Histone H3 staining in the failed mitotic figures.

Aurora B, INCENP (Adams et al. 2001), and Survivin (Wheatley et al. 2001) form the chromosomal passenger complex, which also includes CSC-1 in C. elegans (Romano et al. 2003) and Borealin/Dasra B in humans (Gassmann et al. 2004; Sampath et al. 2004). Aurora B kinase plays a number of roles during mitosis (Carmena and Earnshaw 2003), including phosphorylating Histone H3 on Ser-10 (Giet and Glover 2001) and detecting errors in chromosome attachment in mitosis (Lampson et al. 2004), and performs an essential, but poorly understood, function in cytokinesis. Chromosomal passenger proteins localize to the inner centromere during mitosis and move to the interzonal microtubules, the cleavage furrow, and eventually the midbody during cytokinesis. Because the sequences that targeted *CG4454* and *aurora B* both had 21-bp overlaps with other genes in the dsRNA collection we screened (see Table S3), we remade dsRNA targeting different areas of these two genes and observed no change in phenotype. Since RNAi depletion of the new gene we discovered in our screen, *CG4454,* resulted in the same phenotype as depletion of *aurora B* and *INCENP,* we hypothesized that it could be a new member of the chromosomal passenger complex. We constructed green fluorescent protein (GFP) fusion proteins to both C- and N-termini of CG4454. CG4454-GFP exhibited the signature localization of a passenger protein and co-localized with Aurora B throughout mitosis and cytokinesis (Figure 6), suggesting that it might be complexed to Aurora B. RNAi depletion of *CG4454* or *aurora B* resulted in an absence of phosphorylated Histone H3 on mitotic chromosomes (Figure 7, bottom row), further supporting the participation of CG4454 in the chromosomal passenger complex. Although CG4454 amino acid sequence reveals a remote similarity with Borealin/Dasra B (Gassmann et al. 2004), it is unclear at this point whether CG4454 is its *Drosophila* homolog. Unlike *CG4454,* RNAi depletion of Borealin does not significantly reduce Histone H3 phosphorylation (Gassmann et al. 2004). It might not be possible to confirm whether CG4454 and Borealin are related until structural information becomes available. However, to prevent further confusion in naming conventions, we have decided to tentatively name CG4454 Borealin-related (Borr).

### Detailed Comparison of Binucleine 2 and Aurora B Complex Phenotypes

We compared the phenotypes caused by RNAi depletion of *aurora B* and *borr* to treatment of cells with binucleine 2 using immunofluorescence. The phenotypes were very similar, as judged by perturbation of localization or expression of 14 of the 15 markers used in our detailed analysis (Figure S1), suggesting that the two genes and binucleine 2 perturb a similar step in cytokinesis. The only difference we observed was localization of the chromosomal passenger protein INCENP (Figure 7, top row). No INCENP staining at any cell site was detected in *borr*-depleted cells, suggesting that Borr is required for INCENP localization. This phenotype was also observed in Borealin-depleted cells (Gassmann et al. 2004). In contrast, we observed INCENP accumulations in binucleine 2–treated and *aurora B*–depleted cells. INCENP localizes to the chromosome arms during prometaphase in *aurora B*–depleted cells, which is consistent with reported observations (Adams et al. 2001). In cells exposed to binucleine 2, INCENP aggregated (Figure 7, top row), but did not appear to co-localize with Aurora B or DNA. Given its effect on INCENP localization, binucleine 2 might be a useful tool to study the localization and movement of the Aurora B complex during mitosis and cytokinesis, since the factors that regulate these processes remain obscure. In total, binucleine 2 shares phenotypes with *aurora B* RNAi and affects localization of INCENP, a member of the Aurora B complex, suggesting that binucleine 2 targets the Aurora pathway. Small molecules can target and inhibit protein activity directly, whereas dsRNAs target destruction of mRNA. This difference in mechanism between small molecule inhibition and RNAi could account for the variation in INCENP localization we observed. The specific activity of binucleine 2, however, is very highly related to its structure. We assessed the effect of several similar compounds and found that none were more active than binucleine 2, while most had very little activity (Figure S2).

**Figure 8:**
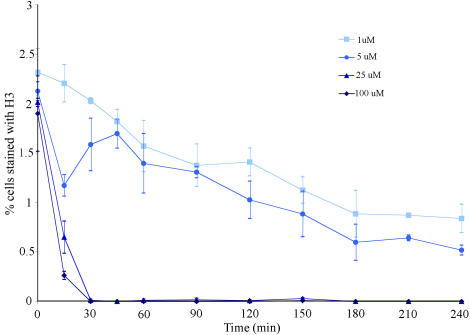
Time- and Concentration-Dependence of Binucleine 2 Kc_167_ cells were treated with 1 μM, 5 μM, 25 μM, or 100 μM binucleine 2. Phospho-Histone H3 staining was assessed at different time points. Binucleine 2 at 100 nM and 300 nM was also tested and showed no effect (data not shown).

To test whether binucleine 2 inhibits Aurora B kinase function, we monitored Histone H3 phosphorylation on Ser-10 in mitotic cells (Giet and Glover 2001). When cells were exposed to binucleine 2, phospho-Histone H3 was absent on chromosomes in mitotic cells (Figure 7, bottom row). To get a quantitative measure of both the concentration of binucleine 2 required and the speed of its action, we assayed about 10,000 cells per time point and concentration for phospho-Histone H3 staining by immunofluorescence (Figure 8). We were unable to detect phospho-Histone H3 in cells treated with 25 μM or 100 μM binucleine 2 for only 30 min, while the percentage of cells exhibiting phospho-Histone H3 staining decreased over time in cells treated with binucleine 2 at 1 μM and 5 μM (Figure 8). While binucleine 2 inhibits Aurora B–dependent phosphorylation, it is not a general kinase inhibitor. Binucleine 2 did not inhibit cyclin-dependent-kinase-dependent entry into mitosis and had no effect on bulk phosphorylation activity in a *Drosophila* cell extract (data not shown). Altogether, phenotypic similarities between loss-of-function for Aurora B and binucleine 2 strongly suggest that binucleine 2 targets a protein involved in the Aurora B pathway. Several small molecule inhibitors of Aurora kinases have been reported, although their chemical scaffolds are different from binucleine 2. These small molecules are not active in fly cells, while binucleine 2 is inactive in mammalian systems (data not shown). Aurora kinase levels are elevated in some tumors, making these proteins a potential target for cancer therapy. Interestingly, lower binucleine 2 concentration or shorter Aurora B RNAi treatment favors binucleate formation, while higher drug concentration and longer incubation for RNAi results in a relative increase in large cells with diffuse DNA. Thus, the cytokinesis function of the Aurora pathway may be more sensitive to inhibition than its mitosis function. This observation may be important for understanding the response of cancer cells to Aurora inhibitors now entering clinical trials (Harrington et al. 2004).

### Conclusion

In summary, our parallel screening approach succeeded in identifying new proteins involved in cytokinesis, and new small molecules that inhibit it. We identified 214 proteins important for cytokinesis, including 25 previously uncharacterized predicted proteins. Depletion of one new gene, *borr,* had a profound effect on cytokinesis. Borr exhibits the signature localization of a chromosomal passenger protein and co-localizes with Aurora B kinase throughout the cell cycle. We also uncovered a potential role of the COPI coatomer complex in cytokinesis. By comparative phenotypic analysis we were able to show that one class of small molecules targets actin cortex integrity, and another the Aurora B pathway. A third class of small molecules, whose phenotype has no RNAi counterpart, presumably causes gain-of-function effects. Traditional methods like affinity chromatography and enzyme inhibition assays will be required to describe the precise biochemical mechanisms of these new cytokinesis inhibitors, but the information already gained from comparative screening will focus this work and allow rapid confirmation or invalidation of candidate biochemical targets.

The problem of target identification has been one of the main barriers to more widespread use of phenotype-based screening in drug discovery. As functional genomic data and systematic RNAi resources become widely available for human cells, parallel screening approaches like the one we describe could be used to discover leads for therapeutic drugs as well as research reagents.

## Materials and Methods

### 

#### Small molecule screen

20,000 *Drosophila* Kc_167_ cells in 40 μl of medium (Schneider's Drosophila Medium [GIBCO, San Diego, California, United States] supplemented with 10% heat-inactivated fetal bovine serum [HyClone, South Logan, Utah, United States] and penicillin/streptomycin [Cellgro, Mediatech, Herdon, Virginia, United States]) were added to each well using a MultiDrop 384 (Thermo Electron, Waltham, Massachusetts, United States) liquid dispenser and incubated at 24 °C overnight. Then, 100 nl of compound stocks dissolved in DMSO at approximately 10 mg/ml was added using the pin transfer robot at the Institute of Chemistry and Cell Biology at Harvard Medical School (http://iccb.med.harvard.edu). Cells were incubated at 24 °C for 48 h. All fixation, staining, and washing steps were carried out using a MultiDrop liquid dispenser and 24-channel wand (V&P Scientific, San Diego, California, United States) for liquid removal. Cells were fixed and permeabilized in 40 μl of 100 mM Pipes/KOH (pH 6.8), 10 mM EGTA, 1 mM MgCl_2_, 3.7% formaldehyde, and 0.2% TritonX-100 for 15 min and washed in 50 μl of PBS. The cytoplasm was stained with 40 μl of 0.5 μg/ml NHS-tetramethylrhodamine (TMR, 5-[and-6]-carboxytetramethylrhodamine, succinimidyl ester C-1171, Molecular Probes, Eugene, Oregon, United States) in PBS for 15 min. Subsequently, 40 μl of 5 μg/ml Hoechst 33342 (Sigma, St. Louis, Missouri, United States) in TBST (TBS with 1% TritonX-100) was added for 30 min. This step stains the DNA and quenches excess NHS ester to ensure uniform TMR staining. Cells were washed twice with 40 μl of TBST and sealed with aluminum seals (Costar 6570, Corning, Corning, New York, United States) for image acquisition.

#### Pyrene–actin assay

Pyrene-labeled actin (2 μM, final concentration; 80 μl, final volume) was added to 10 mM HEPES (pH 7.7), 2 mM MgCl_2_, 100 μM CaCl_2_, 100 mM KCl, 5 mM EGTA, 200 μM ATP, and 10 μM small molecule. Pyrene–actin polymerization was followed by fluorescence spectroscopy over 45 min. An increase in fluorescence indicates actin polymerization. Adapted from Peterson et al. (2001).

#### RNAi screen

dsRNAs were aliquoted into black, clear-bottom 384-well plates (Costar 3712, Corning) at the Drosophila RNAi Screening Center at Harvard Medical School (http://www.flyrnai.org). Each well contained 5 μl of approximately 0.05 μg/μl dsRNA in water. 10,000 *Drosophila* Kc_167_ cells in 10 μl of serum-free Schneiders's Drosophila Medium were added to each well containing dsRNA using a MultiDrop liquid dispenser. After 1 h of incubation at room temperature, 30 μl of medium (Schneider's Drosophila Medium supplemented with 10% heat-inactivated fetal bovine serum and penicillin/streptomycin) was added. The plates were sealed or placed in a humidified chamber and incubated for 4 d at 24 °C. Fixation, TMR, and Hoechst staining were carried out as described above for the small molecule screen. Cells were then blocked in 40 μl of AbDil (TBST with 2% BSA) for 30 min and stained overnight at 4 °C with 20 μl of 1:250 monoclonal anti-tubulin (DM1α, Sigma) and 2 μg/ml Alexa 488 goat anti-mouse antibody (Molecular Probes) in AbDil. Cells were washed twice with 40 μl of TBST and sealed with aluminum seals (Costar 6570) for image acquisition. In order to identify weak hits reliably, the RNAi screen was carried out in triplicate on three separate occasions.

#### Image acquisition

Plates from the RNAi and small molecule screens were imaged using a Universal Imaging (Downingtown, Pennsylvania, Unites States) AutoScope or a Universal Imaging Discovery-1. The AutoScope is a Nikon (Tokyo, Japan) TE300 inverted fluorescence microscope with filter wheel (Lamda10-2, Sutter Instruments, Novato, California, United Stats), x-y stage (Prior H107N300), piezoelectric-motorized objective holder (P-723.10, Physik Instruments, Downingtown, Pennsylvania, United States), and a CCD camera (OrcaER, Hamamatsu, Hamamatsu City, Japan). MetaMorph software (Universal Imaging) running the Screen Acquisition drop-in allowed coordination of software-based autofocusing, movement between wells, imaging, and image evaluation. Two images per well were acquired in each of two (small molecule screen) or three (RNAi screen) channels using a 20x objective with 2 × 2 binning.

#### Scoring of images

Given the need for greater accuracy in an annotation screen, the RNAi images were initially scored by visual inspection. We looked at two images per well from two independent datasets (datasets 2 and 3, approximately 85,000 images) and noted wells with elevated binucleate levels. To determine the percentage of binucleate cells per image, we used the Integrated Morphometry feature in the MetaMorph software to count the number of nuclei per image and then manually counted the number of binucleate cells. We collected two images per assay well and report the level of binucleates per well as an average of both images. Because the level of binucleates can vary depending on the location of the well in the assay plate or the position of the assay plate in a stack of plates, we decided to compare each proposed hit well to its two neighboring wells to prevent false positives or negatives due to local variations. If a neighboring well also exhibited a phenotype, we chose the next neighbor for our analysis. Although we performed the screen in triplicate, we were only able to apply this analysis to two datasets because the cells in the third screen were too clustered to use automated cell counting. It was possible, however, to estimate the binucleate level in the third dataset by visual inspection. We only scored a phenotype if it repeated in at least two experiments, and the vast majority of phenotypes repeated in all three datasets. In a weakly penetrant phenotype the binucleate level was increased by more than 1.25-fold relative to the average of both neighboring wells. In a medium penetrance phenotype the binucleate level was above 4%, or four times as high as the neighboring wells. In a strongly penetrant phenotype the binucleate level was above 15%.

#### Borr-GFP cloning and transfection

For the C-terminal fusion protein, Borr (CG4454) cDNA (LD36125) was cloned into the EcoRI and KpnI sites of pEGFP-C1 (Clontech, Palo Alto, California, United States), cut with NheI and KpnI, and ligated into pPacPL (Krasnow et al. 1989). For the N-terminal fusion, CG4454 digested with SpeI and HindIII, pEGFP-N1 (Clontech) digested with NotI and HindIII, and pPacPL digested with SpeI and NotI were ligated in a triple ligation reaction. Kc_167_ cells were transfected with these constructs using Insect GeneJuice transfection reagent (Novagen, Madison, Wisconsin, United States) according to the manufacturer's instructions and were used for live cell imaging and immunofluorescence 6–7 d after transfection.

#### Immunofluorescence analysis

Cells were exposed to dsRNA or small molecules, fixed, and stained as described in the screening protocols. Cells were stained with TRITC-labeled phalloidin (Sigma) to visualize actin or antibodies to the following proteins (data not shown): Anillin, Aurora B (a gift from D. Glover), Diaphanous, INCENP (a gift from W. Earnshaw), Lamin (a gift from H. Saumweber), Lava-lamp, Myosin II, Pavarotti, Peanut, Pebble (a gift from H. Bellen), phospho-Histone H3 (Upstate Biotechnology, Lake Placid, New York, United States), Polo (a gift from D. Glover), Rho1 (from the Developmental Studies Hybridoma Bank) and tubulin (DM1α, Sigma).

#### Synthesis of *N′*-[1-(3-chloro-4-fluorophenyl)-4-cyano-1H-pyrazol-5-yl]-*N*,*N*-dimethyl iminoformamide (binucleine 2)

Since binucleine 2 is no longer available commercially, we resynthesized it (see Figure S3): 3-chloro-4-fluorophenylhydrazine hydrochloride (compound 1 in Figure S3) (500 mg, 2.5 mmol, Alfa Aesar, Karlsruhe, Germany) and ethoxymethylenemalononitrile (compound 2 in Figure S3) (305 mg, 2.5 mmol, Sigma-Aldrich, St. Louis, Missouri, United States) were refluxed in 3 ml of ethanol for 4 h. The resulting pyrazol (compound 3 in Figure S3) was partially purified by recrystallization from ethanol. Pyrazol (140 mg, 0.5 mmol) and *N,N*-dimethylformamide dimethyl acetal (150 μl, 1 mmol, Aldrich) were refluxed in ethanol for 1 h. The product (binucleine 2) (compound 4 in Figure S3) was recrystallized from ethanol. ^1^H NMR (500 MHz, (CD_3_)_2_SO) δ 8.29 (s, 1 H), 8.06 (dd, J_1_ = 2.7 Hz, J_2_ = 6.8 Hz, 1 H), 8.03 (s, 1 H), 7.84–7.81 (m, 1 H), 7.55 (t, J = 9.2 Hz, 1 H), 3.14 (s, 3 H), 3.00 (s, 3 H). ESI-MS calculated for C_13_H_11_ClFN_5_ 291, [M + H]^+^ found 292.

#### Dose response of binucleine 2.

Kc_167_ cells were treated with 100 nM, 300 nM, 1μM, 5μM, 25μM, or 100 μM of binucleine 2 and fixed after 15 min, 30 min, 45 min, 1 h, 1.5 h, 2 h, 2.5 h, 3 h, 3.5 h, or 4h. After staining with phospho-Histone H3 antibody (Upstate), tubulin (DM1α, Sigma), and DNA and then imaging, cells with phospho-Histone H3 staining were counted. The total number of cells was counted using the Integrated Morphometry feature in the MetaMorph software, and the percentage of cells with H3 staining was calculated and is plotted in Figure 8. Approximately 4,000–5,000 cells per experiment were assessed in two separate experiments for each time point.

## Supporting Information

Figure S1 Examples of Detailed Secondary Analysis Using Immunofluorescence Cells were untreated or treated with binucleine 2 (100 μM, 48 h) or dsRNA corresponding to *aurora B* or *borr (CG4454)*. Cells were stained with TRITC-labeled phalloidin to visualize actin, or antibodies against Anillin, tubulin, Lava-lamp, or Lamin. Lamin-stained cells were treated with binucleine 2 (100 μM) for 24 h.
(4.5 MB TIF).

Figure S2 Structure Activity Relationships for Binucleine 2 (49KB DOC).

Figure S3 Synthesis of Binucleine 2 (32 KB DOC).

Table S1 Small Molecule Phenotypes and Structures Kc_167_ cells were exposed to small molecules at 100 μM, 30 μM, or 10 μM for 48 h. In a weakly penetrant phenotype (w), the binucleate level was increased by at least 1.25-fold above background. In a medium penetrance phenotype (m), the binucleate level was above 4%, and in a strongly penetrant phenotype (s), the binucleate level was above 15%, while the average binucleate level was approximately 1%. In the binucleate phenotype column, “binucleate” indicates binucleate cells only, “diffuse DNA,” binucleate cells with large, diffuse DNA, “lc,” binucleate cells with low cell count, and “MT ext,” binucleate cells with microtubule extensions. HeLa and BSC-1 cells were exposed to small molecules at 30 μM for 24 h. Growth inhibition in drug-sensitive S. cerevisiae RDY98 (Mat a, erg6ΔTRP1^cg^, pdr1ΔKAN, pdr3ΔHIS5+, ade2, trp1, his3, leu2, ura3, can1) was measured at a small molecule concentration of 250 μM after an overnight exposure.
(159 KB DOC).

Table S2 List of Targeted Genes Identified by Binucleate Cells in the RNAi Screen The “DRSC dsRNA ID” is an internal dsRNA ID number. In the potency column, “s” represents strong, “m,” medium and “w,” weak penetrance of the binucleate cell phenotype. In the phenotypic classification column, “binucleate” indicates binucleate cells only, “diffuse DNA,” binucleate cells with large, diffuse DNA, “lc,” binucleate cells with low cell count, and “MT ext,” binucleate cells with microtubule extensions. Six genes were independently identified in multiple wells, either scored twice—*CG10522, cycA, Pp4-19C, RpL32, Tra1,* and *Ubi-63E*—or three times—*crn*.
(141 KB DOC).

Table S3 Information about Genes with Binucleate Phenotypes and Quantitative Analysis of Binucleate Phenotypes Gene names, FlyBase IDs, Gene Ontology annotations, forward and reverse primers, and amplicon lengths are shown. “HFA amplicon” and “DRSC dsRNA ID” are internal dsRNA identifiers. The number of potential secondary targets based on 21 nucleotide fragments is the number of genes that have at least one length of 21 bp or more with matching sequence of 21 bp or more of this amplicon. The criteria used in this analysis are such that it may be prone to false positives for secondary targets. The binucleate percentage per well, the relative increase in binucleates relative to the neighboring wells (1.25 = 25% increase), cell number per well, and relative increase or decrease in cell number relative to the neighboring wells are shown for datasets 1 and 2. Annotations for dataset 3 are only shown when they help to define a particular phenotype.
(97KB XLS).

Table S4 Genes That Scored in the RNAi Screen Sorted by Assigned Functional Groups Functional groups are based on the predicted function as reported by FlyBase (new annotation excluded).
(90 KB DOC).

Table S5 Genes Reported to Be Involved in Cytokinesis and Genes That Resulted in Strong and Medium RNAi Phenotypes (181 KB DOC).

## Abbreviations

Borr: Borealin-related
dsRNA: double-stranded RNA
GFP: green fluorescent protein
RNAi: RNA interference
