# Supplementary figures and images for: Parallel Chemical Genetic and Genome-Wide RNAi Screens Identify Cytokinesis Inhibitors and Targets
Source: PLoS Biol. 2004 Oct 5;2(12):e379. doi: 10.1371/journal.pbio.0020379 (PMC528723; doi:10.1371/journal.pbio.0020379)

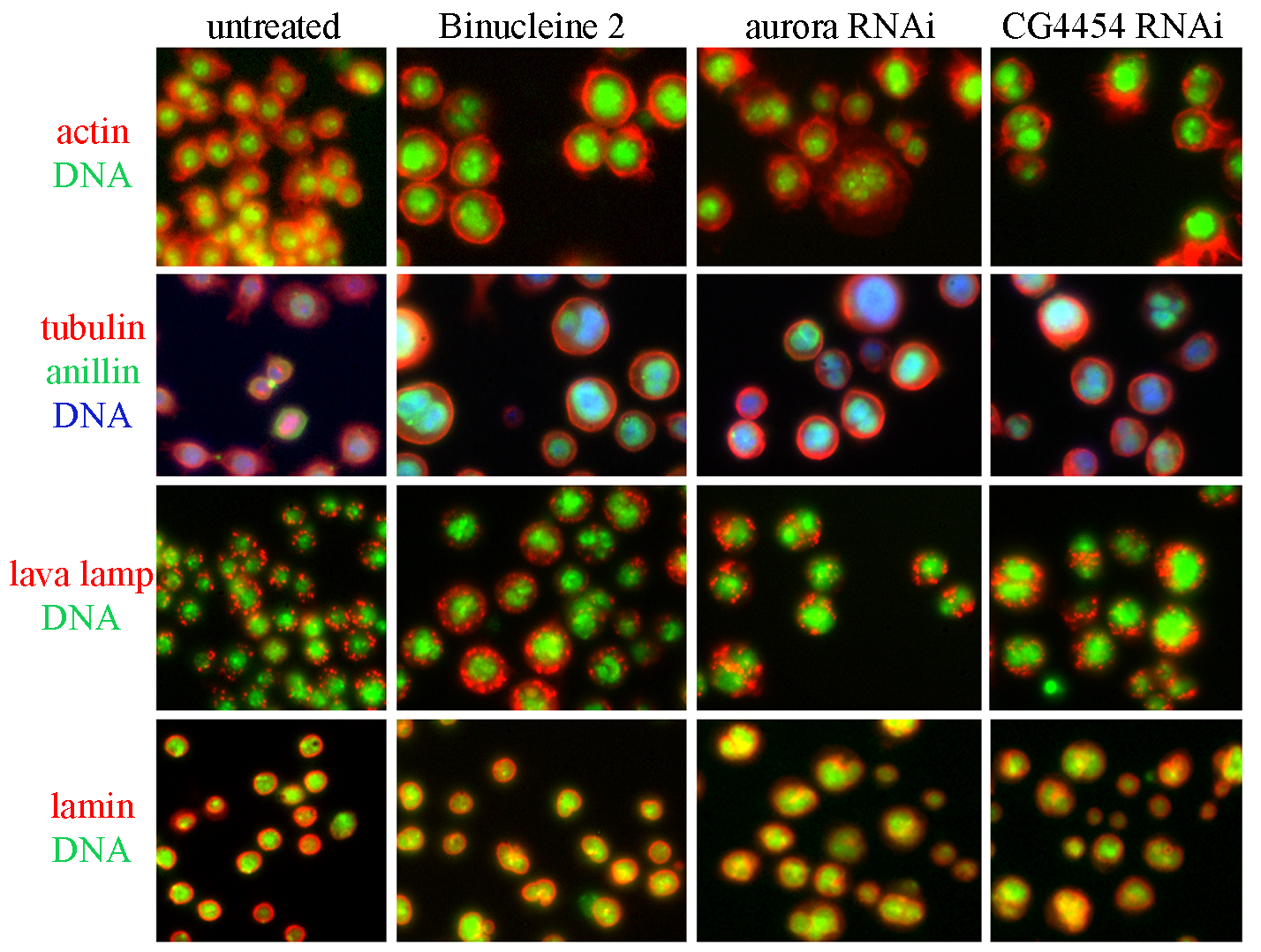

Supplement: Figure S1 — Cells were untreated or treated with binucleine 2 (100 μM, 48 h) or dsRNA corresponding to aurora B or borr (CG4454). Cells were stained with TRITC-labeled phalloidin to visualize actin, or antibodies against Anillin, tubulin, Lava-lamp, or Lamin. Lamin-stained cells were treated with binucleine 2 (100 μM) for 24 h. (4.5 MB TIF). [file pbio.0020379.sg001.tif]
