# Supplementary material for: Parallel Chemical Genetic and Genome-Wide RNAi Screens Identify Cytokinesis Inhibitors and Targets
Source: PLoS Biol. 2004 Oct 5;2(12):e379. doi: 10.1371/journal.pbio.0020379 (PMC528723; doi:10.1371/journal.pbio.0020379)
Supplement: Table S1 — Kc167 cells were exposed to small molecules at 100 μM, 30 μM, or 10 μM for 48 h. In a weakly penetrant phenotype (w), the binucleate level was increased by at least 1.25-fold above background. In a medium penetrance phenotype (m), the binucleate level was above 4%, and in a strongly penetrant phenotype (s), the binucleate level was above 15%, while the average binucleate level was approximately 1%. In the binucleate phenotype column, “binucleate” indicates binucleate cells only, “diffuse DNA,” binucleate cells with large, diffuse DNA, “lc,” binucleate cells with low cell count, and “MT ext,” binucleate cells with microtubule extensions. HeLa and BSC-1 cells were exposed to small molecules at 30 μM for 24 h. Growth inhibition in drug-sensitive S. cerevisiae RDY98 (Mat a, erg6ΔTRP1cg, pdr1ΔKAN, pdr3ΔHIS5+, ade2, trp1, his3, leu2, ura3, can1) was measured at a small molecule concentration of 250 μM after an overnight exposure. (159 KB DOC). [file pbio.0020379.st001.doc]

|  | Kc  100 μM | Kc  30 μM | Kc  10 μM | Binucleate Phenotype | HeLa  30 μM | BSC-1  30 μM | RDY98  250 μM |
| --- | --- | --- | --- | --- | --- | --- | --- |
| Binucleine 1 | w | w | w | binucleate | no | no | no |
| Binucleine 2 | m | m | w | diffuse DNA | no | no | no |
| Binucleine 3 | toxic | m | w | lc | toxic | yes | yes |
| Binucleine 4 | s | s | s | MT ext | yes | yes | no |
| Binucleine 5 | m | m | w | binucleate | yes | yes | no |
| Binucleine 6 | toxic | m | m | binucleate | yes | yes | yes |
| Binucleine 7 | m | m | w | binucleate | yes | yes | no |
| Binucleine 8 | m | w | w | lc | yes | yes | yes |
| Binucleine 9 | m | w | w | MT ext | no | no | no |
| Binucleine 10 | w | m | w | binucleate | no | no | yes |
| Binucleine 11 | toxic | s | m | diffuse DNA | yes | no | no |
| Binucleine 12 | toxic | m | w | binucleate | yes | yes | no |
| Binucleine 13 | toxic | m | m | binucleate | no | no | yes |
| Binucleine 14 | toxic | m | w | binucleate | yes | yes | yes |
| Binucleine 15 | toxic | m | m | lc | toxic | no | no |
| Binucleine 16 | w | w | w | lc | yes | yes | yes |
| Binucleine 17 | w | w | w | MT ext | yes | no | no |
| Binucleine 18 | w | w | w | binucleate | yes | no | no |
| Binucleine 19 | s | s | s | MT ext | yes | yes | yes |
| Binucleine 20 | toxic | w | w | lc | no | no | yes |
| Binucleine 21 | toxic | m | w | lc | no | no | yes |
| Binucleine 22 | toxic | w | w | lc | yes | no | no |
| Binucleine 23 | s | s | s | binucleate | yes | yes | no |
| Binucleine 24 | s | s | s | MT ext | yes | yes | yes |
| Binucleine 25 | s | s | s | MT ext | yes | yes | yes |
