# Supplementary material for: Parallel Chemical Genetic and Genome-Wide RNAi Screens Identify Cytokinesis Inhibitors and Targets
Source: PLoS Biol. 2004 Oct 5;2(12):e379. doi: 10.1371/journal.pbio.0020379 (PMC528723; doi:10.1371/journal.pbio.0020379)
Supplement: Table S2 — The “DRSC dsRNA ID” is an internal dsRNA ID number. In the potency column, “s” represents strong, “m,” medium and “w,” weak penetrance of the binucleate cell phenotype. In the phenotypic classification column, “binucleate” indicates binucleate cells only, “diffuse DNA,” binucleate cells with large, diffuse DNA, “lc,” binucleate cells with low cell count, and “MT ext,” binucleate cells with microtubule extensions. Six genes were independently identified in multiple wells, either scored twice—CG10522, cycA, Pp4-19C, RpL32, Tra1, and Ubi-63E—or three times—crn. (141 KB DOC). [file pbio.0020379.st002.doc]

| Gene | **DRSC dsRNA ID** | | Potency | | **Phenotypic Class** | | Functional Group |
| --- | --- | --- | --- | --- | --- | --- | --- |
| Act57B | DRSC04042 | s | | MT ext | | cytokinesis | |
| Act5C | DRSC17723 | s | | MT ext | | cytokinesis | |
| Act79B | DRSC11604 | m | | MT ext | | cytokinesis | |
| Act87E | DRSC14104 | w | | MT ext | | cytokinesis | |
| Act88F | DRSC14105 | m | | MT ext | | cytokinesis | |
| Arp66B | DRSC09669 | w | | binucleate | | miscellaneous | |
| ATPsyn-beta | DRSC17194 | w | | MT ext | | miscellaneous | |
| ATPsyn-gamma | DRSC14094 | w | | MT ext | | miscellaneous | |
| barr | DRSC03488 | w | | MT ext | | mitosis | |
| betaCop | DRSC20312 | w | | lc | | vesicle transport | |
| beta'Cop | DRSC03492 | m | | lc | | vesicle transport | |
| Bx42 | DRSC17743 | w | | MT ext | | putative domain | |
| CaMKI | DRSC17162 | w | | binucleate | | miscellaneous | |
| cdc2 | DRSC03504 | w | | lc | | mitosis | |
| Cdc27 | DRSC11112 | w | | MT ext | | mitosis | |
| CG10107 | DRSC09698 | w | | binucleate | | putative domain | |
| CG10260 | DRSC18655 | w | | binucleate | | miscellaneous | |
| CG10522 | DRSC09740 | s | | binucleate | | cytokinesis | |
| CG11451 | DRSC11663 | w | | binucleate | | no recognized domain | |
| CG12476 | DRSC03820 | w | | binucleate | | new annotation | |
| CG12972 | DRSC11682 | w | | binucleate | | no recognized domain | |
| CG13028 | DRSC09908 | w | | binucleate | | putative domain | |
| CG1316 | DRSC08304 | w | | binucleate | | translation | |
| CG1340 | DRSC14582 | w | | binucleate | | translation | |
| CG1420 | DRSC14729 | w | | lc, MT ext | | mRNA processing | |
| CG14907 | DRSC15027 | w | | binucleate | | putative domain | |
| CG15157 | DRSC02458 | w | | lc | | no recognized domain | |
| CG15609 | DRSC06561 | w | | binucleate | | putative domain | |
| CG15867 | DRSC02510 | w | | binucleate | | no recognized domain | |
| CG16779 | DRSC15153 | w | | binucleate | | putative domain | |
| CG16941 | DRSC15166 | w | | binucleate | | mRNA processing | |
| CG17137 | DRSC02584 | w | | binucleate | | miscellaneous | |
| CG17905 | DRSC02640 | w | | binucleate | | miscellaneous | |
| CG1796 | DRSC19786 | w | | MT ext | | putative domain | |
| CG18234 | DRSC10309 | w | | binucleate | | putative domain | |
| CG18398 | DRSC02671 | w | | binucleate | | putative domain | |
| CG18591 | DRSC02680 | w | | MT ext | | mRNA processing | |
| CG2807 | DRSC00535 | w | | lc | | mRNA processing | |
| CG31004 | DRSC14472 | w | | binucleate | | putative domain | |
| CG31037 | DRSC15305 | w | | binucleate | | putative domain | |
| CG31284 | DRSC15278 | w | | binucleate | | miscellaneous | |
| CG3203 | DRSC18293 | m | | lc | | ribosomal protein | |
| CG32425 | DRSC11739 | w | | binucleate | | no recognized domain | |
| CG32666 | DRSC19778 | w | | binucleate | | miscellaneous | |
| CG4454 | DRSC02749 | s | | diffuse DNA | | no recognized domain | |
| CG5127 | DRSC15722 | w | | binucleate | | vesicle transport | |
| CG6015 | DRSC15948 | w | | MT ext | | mRNA processing | |
| CG6639 | DRSC02978 | w | | binucleate | | no recognized domain | |
| CG6694 | DRSC10696 | w | | binucleate | | putative domain | |
| CG6749 | DRSC10704 | w | | MT ext | | miscellaneous | |
| CG6800 | DRSC16119 | w | | binucleate | | cell cycle | |
| CG6885 | DRSC10740 | w | | binucleate | | putative domain | |
| CG7236 | DRSC03037 | w | | binucleate | | cell cycle | |
| CG7552 | DRSC16257 | w | | binucleate | | putative domain | |
| CG7730 | DRSC10909 | w | | binucleate | | no recognized domain | |
| CG8405 | DRSC05599 | w | | binucleate | | no recognized domain | |
| CG8636 | DRSC18427 | w | | lc | | translation | |
| Chc | DRSC20229 | w | | binucleate | | vesicle transport | |
| cpb | DRSC00809 | w | | binucleate | | miscellaneous | |
| crn | DRSC18755 | m | | lc, MT ext | | mRNA processing | |
| CycA | DRSC11123 | w | | lc, diffuse DNA | | cell cycle | |
| deltaCOP | DRSC18760 | m | | lc | | vesicle transport | |
| dia | DRSC03519 | s | | binucleate | | cytokinesis | |
| dm | DRSC18762 | w | | lc | | transcription | |
| dmt | DRSC16932 | w | | MT ext | | no recognized domain | |
| Dox-A2 | DRSC03318 | w | | MT ext | | proteasome endopeptidase | |
| Dp | DRSC07402 | m | | lc | | transcription | |
| E(Pc) | DRSC05281 | w | | binucleate | | miscellaneous | |
| E2f | DRSC16655 | w | | lc | | transcription | |
| eIF-3p66 | DRSC16938 | w | | binucleate | | translation | |
| eIF3-S10 | DRSC12339 | w | | lc | | translation | |
| eIF3-S8 | DRSC06905 | w | | binucleate | | translation | |
| feo | DRSC19398 | s | | diffuse DNA | | cytokinesis | |
| fwd | DRSC08580 | w | | binucleate | | cytokinesis | |
| gammaCop | DRSC16955 | m | | lc | | vesicle transport | |
| geminin | DRSC04984 | m | | lc, diffuse DNA | | cell cycle | |
| hbn | DRSC04082 | w | | binucleate | | transcription | |
| His2B:CG17949 | DRSC21265 | w | | MT ext | | no recognized domain | |
| His3:CG31613 | DRSC21267 | w | | lc, MT ext | | miscellaneous | |
| His4r | DRSC16703 | m | | lc, MT ext | | miscellaneous | |
| hoip | DRSC03546 | m | | lc, MT ext | | mRNA processing | |
| Hrb27C | DRSC03347 | w | | binucleate | | mRNA processing | |
| Hsc70-4 | DRSC16711 | w | | binucleate | | vesicle transport | |
| ial | DRSC03548 | s | | diffuse DNA | | mitosis | |
| l(2)03709 | DRSC06512 | w | | binucleate | | miscellaneous | |
| lace | DRSC03564 | w | | binucleate | | miscellaneous | |
| Lip2 | DRSC02578 | w | | binucleate | | miscellaneous | |
| lola | DRSC05220 | w | | binucleate | | transcription | |
| maf-S | DRSC07348 | w | | binucleate | | transcription | |
| Mes2 | DRSC11593 | w | | binucleate | | no recognized domain | |
| mor | DRSC15378 | w | | MT ext | | transcription | |
| Mov34 | DRSC04624 | w | | MT ext | | proteasome endopeptidase | |
| msi | DRSC17003 | w | | binucleate | | translation | |
| mts | DRSC03574 | w | | MT ext | | miscellaneous | |
| ncd | DRSC17012 | m | | diffuse DNA | | mitosis | |
| noi | DRSC12383 | w | | binucleate | | mRNA processing | |
| pAbp | DRSC07659 | w | | binucleate | | translation | |
| Pabp2 | DRSC07501 | m | | lc | | translation | |
| pav | DRSC08730 | s | | binucleate | | cytokinesis | |
| pbl | DRSC11381 | s | | binucleate | | cytokinesis | |
| Pka-C2 | DRSC16790 | m | | diffuse DNA | | miscellaneous | |
| Pp4-19C | DRSC20559 | w | | binucleate | | mitosis | |
| Pros35 | DRSC03401 | w | | MT ext | | proteasome endopeptidase | |
| Qm | DRSC11947 | w | | lc | | ribosomal protein | |
| Rab1 | DRSC16808 | w | | binucleate | | vesicle transport | |
| RacGAP50C | DRSC07575 | s | | binucleate | | cytokinesis | |
| Rca1 | DRSC03407 | w | | lc, diffuse DNA | | cell cycle | |
| rdh | DRSC08434 | w | | binucleate | | no recognized domain | |
| Rho1 | DRSC07530 | s | | binucleate | | cytokinesis | |
| RhoL | DRSC16824 | w | | binucleate | | miscellaneous | |
| RnrL | DRSC03413 | w | | binucleate | | miscellaneous | |
| RnrS | DRSC07533 | w | | lc | | miscellaneous | |
| rok | DRSC20277 | w | | binucleate | | cytokinesis | |
| Rpb5 | DRSC06100 | w | | binucleate | | transcription | |
| RpII215 | DRSC20280 | m | | lc | | transcription | |
| RpL10Ab | DRSC10798 | m | | lc | | ribosomal protein | |
| RpL11 | DRSC07537 | m | | lc | | ribosomal protein | |
| RpL12 | DRSC04344 | m | | lc | | ribosomal protein | |
| RpL14 | DRSC11269 | w | | lc | | ribosomal protein | |
| RpL15 | DRSC20963 | w | | lc | | ribosomal protein | |
| RpL18A | DRSC07538 | m | | lc | | ribosomal protein | |
| RpL19 | DRSC04649 | m | | lc | | ribosomal protein | |
| RpL21 | DRSC03704 | w | | lc | | ribosomal protein | |
| RpL22 | DRSC18707 | m | | lc | | ribosomal protein | |
| RpL23 | DRSC04648 | w | | lc | | ribosomal protein | |
| RpL26 | DRSC10726 | w | | lc, MT ext | | ribosomal protein | |
| RpL27 | DRSC15638 | w | | lc | | ribosomal protein | |
| RpL27A | DRSC00781 | m | | lc | | ribosomal protein | |
| RpL3 | DRSC16834 | m | | lc | | ribosomal protein | |
| RpL31 | DRSC06716 | m | | binucleate | | ribosomal protein | |
| RpL32 | DRSC16835 | m | | lc | | ribosomal protein | |
| RpL35 | DRSC18347 | m | | lc | | ribosomal protein | |
| RpL35A | DRSC12302 | w | | binucleate | | ribosomal protein | |
| RpL36 | DRSC18708 | w | | lc | | ribosomal protein | |
| RpL36A | DRSC03055 | w | | binucleate | | ribosomal protein | |
| RpL39 | DRSC04651 | w | | binucleate | | ribosomal protein | |
| RpL4 | DRSC16833 | w | | lc | | ribosomal protein | |
| RpL7 | DRSC03417 | m | | lc | | ribosomal protein | |
| RpL7A | DRSC18709 | w | | lc | | ribosomal protein | |
| RpL8 | DRSC08695 | w | | binucleate | | ribosomal protein | |
| RpL9 | DRSC03418 | m | | lc | | ribosomal protein | |
| Rpn1 | DRSC11274 | w | | MT ext | | proteasome endopeptidase | |
| Rpn2 | DRSC16839 | w | | lc, MT ext | | proteasome endopeptidase | |
| Rpn6 | DRSC07541 | m | | lc, MT ext | | proteasome endopeptidase | |
| Rpn7 | DRSC16841 | w | | lc, MT ext | | proteasome endopeptidase | |
| RpS12 | DRSC11270 | w | | lc | | ribosomal protein | |
| RpS13 | DRSC03419 | m | | lc | | ribosomal protein | |
| RpS14a | DRSC18710 | w | | lc | | ribosomal protein | |
| RpS14b | DRSC18711 | m | | lc | | ribosomal protein | |
| RpS15Ab | DRSC06129 | m | | lc | | ribosomal protein | |
| RpS16 | DRSC04442 | w | | binucleate | | ribosomal protein | |
| RpS18 | DRSC07540 | w | | binucleate | | ribosomal protein | |
| RpS19a | DRSC20281 | m | | lc | | ribosomal protein | |
| RpS24 | DRSC04414 | w | | binucleate | | ribosomal protein | |
| RpS26 | DRSC03420 | m | | lc | | ribosomal protein | |
| RpS27 | DRSC14244 | w | | binucleate | | ribosomal protein | |
| RpS29 | DRSC16433 | w | | lc | | ribosomal protein | |
| RpS3 | DRSC16838 | w | | lc | | ribosomal protein | |
| RpS30 | DRSC15119 | m | | lc | | ribosomal protein | |
| RpS3A | DRSC17168 | w | | lc | | ribosomal protein | |
| RpS4 | DRSC11272 | m | | lc | | ribosomal protein | |
| RpS6 | DRSC18712 | m | | lc | | ribosomal protein | |
| RpS7 | DRSC15394 | m | | lc | | ribosomal protein | |
| RpS8 | DRSC16318 | m | | lc | | ribosomal protein | |
| RpS9 | DRSC11273 | w | | lc | | ribosomal protein | |
| sbr | DRSC20368 | w | | MT ext | | mRNA processing | |
| scra | DRSC07679 | s | | binucleate | | cytokinesis | |
| shi | DRSC20373 | m | | binucleate | | vesicle transport | |
| Slh | DRSC00789 | w | | binucleate | | vesicle transport | |
| Smr | DRSC19495 | w | | lc | | transcription | |
| smt3 | DRSC03611 | w | | lc, MT ext | | miscellaneous | |
| Snap | DRSC11285 | w | | binucleate | | miscellaneous | |
| snRNP69D | DRSC09800 | w | | lc | | mRNA processing | |
| Spx | DRSC18720 | w | | MT ext | | mRNA processing | |
| sqh | DRSC18837 | w | | binucleate | | cytokinesis | |
| stan | DRSC05234 | w | | binucleate | | miscellaneous | |
| stg | DRSC17071 | w | | binucleate | | cell cycle | |
| Su(var)3-9 | DRSC13081 | w | | lc | | translation | |
| sws | DRSC18843 | w | | binucleate | | putative domain | |
| Syx5 | DRSC03432 | m | | binucleate | | vesicle transport | |
| Tra1 | DRSC04884 | w | | binucleate | | miscellaneous | |
| Trap170 | DRSC08235 | w | | lc | | transcription | |
| tsr | DRSC04718 | m | | binucleate | | cytokinesis | |
| twit | DRSC18575 | w | | binucleate | | no recognized domain | |
| Ubi-p63E | DRSC08703 | w | | lc | | miscellaneous | |
| Wnt4 | DRSC00976 | m | | binucleate | | miscellaneous | |
| yip6 | DRSC03801 | w | | lc | | ribosomal protein | |
| zetaCOP | DRSC11412 | w | | lc | | vesicle transport | |
| zip | DRSC04725 | s | | binucleate | | cytokinesis | |
|  | DRSC19060 | w | | binucleate | | miscellaneous | |
|  | DRSC01059 | w | | binucleate | | new annotation | |
|  | DRSC01398 | w | | binucleate | | new annotation | |
|  | DRSC03975 | w | | binucleate | | new annotation | |
|  | DRSC03998 | w | | binucleate | | new annotation | |
|  | DRSC05059 | w | | binucleate | | new annotation | |
|  | DRSC05209 | w | | MT ext | | new annotation | |
|  | DRSC05316 | w | | binucleate | | new annotation | |
|  | DRSC05734 | w | | binucleate | | new annotation | |
|  | DRSC07773 | w | | binucleate | | new annotation | |
|  | DRSC07932 | w | | binucleate | | new annotation | |
|  | DRSC07960 | w | | binucleate | | new annotation | |
|  | DRSC08022 | w | | binucleate | | new annotation | |
|  | DRSC09009 | w | | binucleate | | new annotation | |
|  | DRSC09064 | w | | binucleate | | new annotation | |
|  | DRSC09069 | w | | binucleate | | new annotation | |
|  | DRSC09076 | w | | binucleate | | new annotation | |
|  | DRSC09272 | w | | binucleate | | new annotation | |
|  | DRSC09354 | w | | binucleate | | new annotation | |
|  | DRSC12424 | w | | binucleate | | new annotation | |
|  | DRSC13174 | w | | lc | | new annotation | |
|  | DRSC13791 | w | | lc | | new annotation | |
|  | DRSC17650 | w | | binucleate | | new annotation | |
|  | DRSC19272 | w | | binucleate | | new annotation | |
|  | DRSC19287 | w | | binucleate | | new annotation | |
