# Supplementary material for: Parallel Chemical Genetic and Genome-Wide RNAi Screens Identify Cytokinesis Inhibitors and Targets
Source: PLoS Biol. 2004 Oct 5;2(12):e379. doi: 10.1371/journal.pbio.0020379 (PMC528723; doi:10.1371/journal.pbio.0020379)
Supplement: Table S4 — Functional groups are based on the predicted function as reported by FlyBase (new annotation excluded). (90 KB DOC). [file pbio.0020379.st004.doc]

| Gene | | DRSC ID | | Functional Group | | Predicted Function | |
| --- | --- | --- | --- | --- | --- | --- | --- |
| CG6800 | DRSC16119 | | cell cycle | | kinase cdk | |  |
| CG7236 | DRSC03037 | | cell cycle | | kinase cdk | |  |
| CycA | DRSC04984 | | cell cycle | | cyclin | |  |
| geminin | DRSC03407 | | cell cycle | | cell cycle regulator | |  |
| Rca1 | DRSC17071 | | cell cycle | | cell cycle | |  |
| stg | DRSC11123 | | cell cycle | | cell cycle phosphatase | |  |
| Act57B | DRSC07679 | | cytokinesis | | actin | |  |
| Act5C | DRSC20277 | | cytokinesis | | actin | |  |
| Act79B | DRSC18837 | | cytokinesis | | actin | |  |
| Act87E | DRSC04718 | | cytokinesis | | actin | |  |
| Act88F | DRSC04725 | | cytokinesis | | actin | |  |
| CG10522 | DRSC04042 | | cytokinesis | | citron kinase | |  |
| dia | DRSC11604 | | cytokinesis | | formin | |  |
| feo | DRSC16932 | | cytokinesis | | PRC1 | |  |
| fwd | DRSC08580 | | cytokinesis | | 1-phosphatidylinositol 4-kinase | |  |
| pav | DRSC14104 | | cytokinesis | | kinesin | |  |
| pbl | DRSC14105 | | cytokinesis | | rho GEF | |  |
| RacGAP50C | DRSC09740 | | cytokinesis | | rho GAP | |  |
| Rho1 | DRSC00809 | | cytokinesis | | rho GTPase | |  |
| rok | DRSC03519 | | cytokinesis | | rho kinase | |  |
| scra | DRSC08730 | | cytokinesis | | anillin | |  |
| sqh | DRSC11381 | | cytokinesis | | myosin II light chain | |  |
| tsr | DRSC07575 | | cytokinesis | | cofilin | |  |
| zip | DRSC07530 | | cytokinesis | | myosin II heavy chain | |  |
| Arp66B | DRSC09669 | | miscellaneous | | actin binding | |  |
| ATPsyn-b | DRSC05234 | | miscellaneous | | ATP synthase-beta | |  |
| ATPsyn-g | DRSC04884 | | miscellaneous | | ATP synthase-gamma chain | |  |
| CaMKI | DRSC17194 | | miscellaneous | | kinase | |  |
| CG10260 | DRSC18655 | | miscellaneous | | 1-phosphatidylinositol 4-kinase | |  |
| CG11963 | DRSC14094 | | miscellaneous | | succinate-CoA ligase | |  |
| CG17137 | DRSC17162 | | miscellaneous | | voltage-dependent ion-selective channel activity | |  |
| CG17905 | DRSC14446 | | miscellaneous | | LDL receptor | |  |
| CG31284 | DRSC02584 | | miscellaneous | | calcium channel | |  |
| CG32666 | DRSC02640 | | miscellaneous | | kinase | |  |
| CG6749 | DRSC08703 | | miscellaneous | | G-protein coupled receptor activity | |  |
| cpb | DRSC17723 | | miscellaneous | | actin binding | |  |
| E(Pc) | DRSC15278 | | miscellaneous | | establishment and/or maintenance of chromatin architecture | |  |
| His3:CG31613 | DRSC03413 | | miscellaneous | | DNA binding | |  |
| His4r | DRSC00976 | | miscellaneous | | Histone H4 replacement | |  |
| l(2)03709 | DRSC10704 | | miscellaneous | | prohibitin | |  |
| lace | DRSC05281 | | miscellaneous | | serine C-palmitoyltransferase | |  |
| Lip2 | DRSC21267 | | miscellaneous | | triacylglycerol lipase activity | |  |
| mts | DRSC19060 | | miscellaneous | | protein phosphatase type 2A | |  |
| Pka-C2 | DRSC03611 | | miscellaneous | | protein kinase A | |  |
| pll | DRSC03550 | | miscellaneous | | kinase | |  |
| RhoL | DRSC06512 | | miscellaneous | | rho-like GTPase | |  |
| RnrL | DRSC03564 | | miscellaneous | | ribonucleoside-diphosphate reductase | |  |
| RnrS | DRSC16790 | | miscellaneous | | ribonucleoside-diphosphate reductase | |  |
| smt3 | DRSC07533 | | miscellaneous | | ubiquitin-like | |  |
| stan | DRSC17026 | | miscellaneous | | G protein coupled receptor activity | |  |
| Tra1 | DRSC02578 | | miscellaneous | | kinase | |  |
| Ubi-p63E | DRSC16824 | | miscellaneous | | ubiquitin | |  |
| Wnt4 | DRSC03574 | | miscellaneous | | signal transducer activity | |  |
|  | DRSC19904 | | miscellaneous | | transposable element | |  |
| barr | DRSC20559 | | mitosis | | condensin | |  |
| cdc2 | DRSC03504 | | mitosis | | cyclin-dependent kinase | |  |
| Cdc27 | DRSC11112 | | mitosis | | cell cycle mitosis | |  |
| ial | DRSC03548 | | mitosis | | aurora kinase | |  |
| ncd | DRSC17012 | | mitosis | | kinesin | |  |
| Pp4-19C | DRSC03488 | | mitosis | | phosphatase | |  |
| CG1420 | DRSC18755 | | mRNA processing | | pre-mRNA splicing factor activity | |  |
| CG16941 | DRSC14729 | | mRNA processing | | pre-mRNA splicing factor activity | |  |
| CG18591 | DRSC03347 | | mRNA processing | | pre-mRNA splicing factor activity | |  |
| CG2807 | DRSC00535 | | mRNA processing | | pre-mRNA splicing factor activity | |  |
| CG6015 | DRSC12383 | | mRNA processing | | pre-mRNA splicing factor activity | |  |
| crn | DRSC20368 | | mRNA processing | | pre-mRNA splicing factor activity | |  |
| hoip | DRSC09800 | | mRNA processing | | small nuclear ribonucleoprotein complex | |  |
| Hrb27C | DRSC15166 | | mRNA processing | | mRNA processing | |  |
| noi | DRSC02680 | | mRNA processing | | pre-mRNA splicing factor activity | |  |
| sbr | DRSC18720 | | mRNA processing | | mRNA-nucleus export poly(A)+ mRNA-nucleus export | |  |
| snRNP69D | DRSC15948 | | mRNA processing | | small nuclear ribonucleoprotein | |  |
| Spx | DRSC03546 | | mRNA processing | | pre-mRNA splicing factor activity | |  |
| CG11451 | DRSC02510 | | no recognized domain | | unknown function | |  |
| CG12972 | DRSC11663 | | no recognized domain | | unknown function | |  |
| CG13028 | DRSC09908 | | no recognized domain | | unknown function | |  |
| CG13596 | DRSC11682 | | no recognized domain | | unknown function | |  |
| CG14210 | DRSC19398 | | no recognized domain | | unknown function | |  |
| CG15157 | DRSC02749 | | no recognized domain | | unknown function | |  |
| CG15867 | DRSC04231 | | no recognized domain | | unknown function | |  |
| CG4454 | DRSC10909 | | no recognized domain | | unknown function | |  |
| CG6639 | DRSC19566 | | no recognized domain | | unknown function | |  |
| CG7730 | DRSC02978 | | no recognized domain | | unknown function | |  |
| dmt | DRSC21265 | | no recognized domain | | unknown function | |  |
| His2B:CG17949 | DRSC11593 | | no recognized domain | | unknown function | |  |
| Mes2 | DRSC02458 | | no recognized domain | | unknown function | |  |
| rdh | DRSC08434 | | no recognized domain | | unknown function | |  |
| twit | DRSC18575 | | no recognized domain | | unknown function | |  |
| Dox-A2 | DRSC03318 | | proteasome endopeptidase | | diphenol oxidase, proteasome endopeptidase | |  |
| Mov34 | DRSC16839 | | proteasome endopeptidase | | proteasome endopeptidase activity | |  |
| Pros35 | DRSC04624 | | proteasome endopeptidase | | proteasome endopeptidase activity | |  |
| Rpn1 | DRSC07541 | | proteasome endopeptidase | | proteasome endopeptidase activity | |  |
| Rpn2 | DRSC03401 | | proteasome endopeptidase | | proteasome endopeptidase activity | |  |
| Rpn6 | DRSC16841 | | proteasome endopeptidase | | proteasome endopeptidase activity | |  |
| Rpn7 | DRSC11274 | | proteasome endopeptidase | | proteasome endopeptidase activity | |  |
| Bx42 | DRSC16257 | | putative domain | | nuclear SKIP-SNW | |  |
| CG10107 | DRSC17743 | | putative domain | | cysteine-type peptidase activity | |  |
| CG14907 | DRSC09698 | | putative domain | | alpha/beta-Hydrolases | |  |
| CG15609 | DRSC06561 | | putative domain | | calponin-homology domain | |  |
| CG16779 | DRSC15027 | | putative domain | | zinc finger | |  |
| CG1796 | DRSC18843 | | putative domain | | WD40 | |  |
| CG18234 | DRSC15153 | | putative domain | | prolyl 4-hydroxylase alpha subunit C-terminus | |  |
| CG18398 | DRSC19786 | | putative domain | | ARM | |  |
| CG31004 | DRSC10309 | | putative domain | | extracellular domain in nidogen | |  |
| CG31037 | DRSC02671 | | putative domain | | RCC1 | |  |
| CG6694 | DRSC15305 | | putative domain | | zinc finger | |  |
| CG6885 | DRSC18437 | | putative domain | | zinc finger | |  |
| CG7552 | DRSC10740 | | putative domain | | WW domain | |  |
| sws | DRSC10696 | | putative domain | | hydrolase | |  |
| CG3203 | DRSC03704 | | ribosomal protein | | ribosomal protein | |  |
| Qm | DRSC04648 | | ribosomal protein | | ribosomal protein | |  |
| RpL10Ab | DRSC10726 | | ribosomal protein | | ribosomal protein | |  |
| RpL11 | DRSC15638 | | ribosomal protein | | ribosomal protein | |  |
| RpL12 | DRSC00781 | | ribosomal protein | | ribosomal protein | |  |
| RpL14 | DRSC16834 | | ribosomal protein | | ribosomal protein | |  |
| RpL15 | DRSC06716 | | ribosomal protein | | ribosomal protein | |  |
| RpL18A | DRSC06129 | | ribosomal protein | | ribosomal protein | |  |
| RpL19 | DRSC16835 | | ribosomal protein | | ribosomal protein | |  |
| RpL21 | DRSC18347 | | ribosomal protein | | ribosomal protein | |  |
| RpL22 | DRSC12302 | | ribosomal protein | | ribosomal protein | |  |
| RpL23 | DRSC18708 | | ribosomal protein | | ribosomal protein | |  |
| RpL26 | DRSC03801 | | ribosomal protein | | ribosomal protein | |  |
| RpL27 | DRSC03055 | | ribosomal protein | | ribosomal protein | |  |
| RpL27A | DRSC04651 | | ribosomal protein | | ribosomal protein | |  |
| RpL3 | DRSC18707 | | ribosomal protein | | ribosomal protein | |  |
| RpL31 | DRSC16833 | | ribosomal protein | | ribosomal protein | |  |
| RpL32 | DRSC00782 | | ribosomal protein | | ribosomal protein | |  |
| RpL35 | DRSC03417 | | ribosomal protein | | ribosomal protein | |  |
| RpL35A | DRSC18709 | | ribosomal protein | | ribosomal protein | |  |
| RpL36 | DRSC08695 | | ribosomal protein | | ribosomal protein | |  |
| RpL36A | DRSC18293 | | ribosomal protein | | ribosomal protein | |  |
| RpL39 | DRSC11947 | | ribosomal protein | | ribosomal protein | |  |
| RpL4 | DRSC03418 | | ribosomal protein | | ribosomal protein | |  |
| RpL7 | DRSC03419 | | ribosomal protein | | ribosomal protein | |  |
| RpL7A | DRSC18710 | | ribosomal protein | | ribosomal protein | |  |
| RpL8 | DRSC10798 | | ribosomal protein | | ribosomal protein | |  |
| RpL9 | DRSC07537 | | ribosomal protein | | ribosomal protein | |  |
| RpS12 | DRSC18711 | | ribosomal protein | | ribosomal protein | |  |
| RpS13 | DRSC04442 | | ribosomal protein | | ribosomal protein | |  |
| RpS14a | DRSC07540 | | ribosomal protein | | ribosomal protein | |  |
| RpS14b | DRSC20281 | | ribosomal protein | | ribosomal protein | |  |
| RpS15Ab | DRSC06129 | | ribosomal protein | | ribosomal protein | |  |
| RpS16 | DRSC11269 | | ribosomal protein | | ribosomal protein | |  |
| RpS18 | DRSC04414 | | ribosomal protein | | ribosomal protein | |  |
| RpS19a | DRSC03420 | | ribosomal protein | | ribosomal protein | |  |
| RpS24 | DRSC14244 | | ribosomal protein | | ribosomal protein | |  |
| RpS26 | DRSC16433 | | ribosomal protein | | ribosomal protein | |  |
| RpS27 | DRSC20963 | | ribosomal protein | | ribosomal protein | |  |
| RpS29 | DRSC16838 | | ribosomal protein | | ribosomal protein | |  |
| RpS3 | DRSC15119 | | ribosomal protein | | ribosomal protein | |  |
| RpS30 | DRSC07538 | | ribosomal protein | | ribosomal protein | |  |
| RpS3A | DRSC17168 | | ribosomal protein | | ribosomal protein | |  |
| RpS4 | DRSC11272 | | ribosomal protein | | ribosomal protein | |  |
| RpS6 | DRSC18712 | | ribosomal protein | | ribosomal protein | |  |
| RpS7 | DRSC15394 | | ribosomal protein | | ribosomal protein | |  |
| RpS8 | DRSC16318 | | ribosomal protein | | ribosomal protein | |  |
| RpS9 | DRSC11273 | | ribosomal protein | | ribosomal protein | |  |
| yip6 | DRSC04649 | | ribosomal protein | | ribosomal protein | |  |
| dm | DRSC07348 | | transcription | | transcription factor | |  |
| Dp | DRSC18762 | | transcription | | cell cycle transcription factor | |  |
| E2f | DRSC15378 | | transcription | | RNA polymerase II transcription factor activity | |  |
| hbn | DRSC06100 | | transcription | | transcription factor | |  |
| lola | DRSC07402 | | transcription | | RNA polymerase II transcription factor activity | |  |
| maf-S | DRSC16655 | | transcription | | transcription factor activity | |  |
| mor | DRSC08235 | | transcription | | general RNA polymerase II transcription factor activity | |  |
| Rpb5 | DRSC04082 | | transcription | | DNA-directed RNA polymerase activity | |  |
| RpII215 | DRSC05220 | | transcription | | RNA polymerase II | |  |
| Smr | DRSC20280 | | transcription | | transcription co-repressor activity | |  |
| Trap170 | DRSC19495 | | transcription | | RNA polymerase II transcription mediator activity | |  |
| CG1316 | DRSC08304 | | translation | | RNA binding | |  |
| CG1340 | DRSC14582 | | translation | | translation initiation factor activity | |  |
| CG8636 | DRSC17003 | | translation | | translation initiation factor activity | |  |
| eIF-3p66 | DRSC18427 | | translation | | translation initiation factor activity | |  |
| eIF3-S10 | DRSC07659 | | translation | | translation initiation factor activity | |  |
| eIF3-S8 | DRSC16938 | | translation | | translation initiation factor activity | |  |
| msi | DRSC12339 | | translation | | RNA binding | |  |
| pAbp | DRSC06905 | | translation | | polyA-binding protein | |  |
| Pabp2 | DRSC07501 | | translation | | polyA-binding protein | |  |
| Su(var)3-9 | DRSC13081 | | translation | | translation initiation | |  |
| betaCop | DRSC20312 | | vesicle transport | | COPI | |  |
| beta'Cop | DRSC03492 | | vesicle transport | | COPI | |  |
| CG5127 | DRSC15722 | | vesicle transport | | vesicle-mediated transport sec1 | |  |
| Chc | DRSC20229 | | vesicle transport | | clathrin | |  |
| deltaCOP | DRSC00789 | | vesicle transport | | COPI | |  |
| gammaCop | DRSC18760 | | vesicle transport | | COPI | |  |
| Hsc70-4 | DRSC03432 | | vesicle transport | | heat shock -endocytosis | |  |
| Rab1 | DRSC16955 | | vesicle transport | | rab GTPase | |  |
| shi | DRSC16711 | | vesicle transport | | dynamin | |  |
| Slh | DRSC16808 | | vesicle transport | | SNARE binding | |  |
| Syx5 | DRSC20373 | | vesicle transport | | syntaxin5 | |  |
| zetaCOP | DRSC11412 | | vesicle transport | | COPI | |  |
