# Supplementary material for: Parallel Chemical Genetic and Genome-Wide RNAi Screens Identify Cytokinesis Inhibitors and Targets
Source: PLoS Biol. 2004 Oct 5;2(12):e379. doi: 10.1371/journal.pbio.0020379 (PMC528723; doi:10.1371/journal.pbio.0020379)
Supplement: Table S5 — (181 KB DOC). [file pbio.0020379.st005.doc]

| Gene | DRSC ID | Potency | Known Cytokinesis? | | Function and  Reference | Cytokinesis RNAi Phenotype  Reported? |
| --- | --- | --- | --- | --- | --- | --- |
| Act57B  Act5C | DRSC14105 | strong | yes | Actin (Schroeder 1973) | |  |
| CG10522 | DRSC09740 | strong | yes | Citron kinase  (Madaule et al. 1998) | | (Kiger et al. 2003; Rogers et al. 2003) |
| CG4454 | DRSC02749 | strong |  | this work | |  |
| dia | DRSC03519 | strong | yes | Formin (Castrillon and Wasserman 1994; Watanabe et al. 1997) | | (Kiger et al. 2003; Rogers et al. 2003) |
| feo | DRSC19398 | strong | yes | PRC1 (Verni et al. 2004) | | (Verni et al. 2004) |
| ial | DRSC03548 | strong | yes | Aurora B/AIM-1 kinase  (Terada et al. 1998) | | (Kiger et al. 2003) |
| pav | DRSC08730 | strong | yes | MKLP1/CHO1 kinesin  (Nislow et al. 1992; Adams et al. 1998) | | (Somma et al. 2002; Goshima and Vale 2003; Kiger et al. 2003) |
| pbl | DRSC11381 | strong | yes | Rho GEF (Prokopenko et al. 1999; Somers and Saint 2003) | | (Somma et al. 2002; Kiger et al. 2003) |
| RacGAP50C | DRSC07575 | strong | yes | Rho GAP (Hirose et al. 2001; Somers and Saint 2003) | | (Somma et al. 2002; Rogers et al. 2003) |
| Rho1 | DRSC07530 | strong | yes | Rho GTPase (Mabuchi et al. 1993; Prokopenko et al. 1999) | | (Somma et al. 2002; Kiger et al. 2003; Rogers et al. 2003) |
| scra | DRSC07679 | strong | yes | Anillin (Oegema et al. 2000; Somma et al. 2002) | | (Somma et al. 2002; Kiger et al. 2003; Rogers et al. 2003) |
| zip | DRSC04725 | strong | yes | Myosin II heavy chain (Mabuchi and Okuno 1977; De Lozanne and Spudich 1987; Knecht and Loomis 1987) | | (Rogers et al. 2003) |
| Act79B | DRSC04042 | medium | yes | Actin (Schroeder 1973) | |  |
| Ac88F | DRSC17723 | medium | yes | Actin (Schroeder 1973) | |  |
| Beta’COP | DRSC03492 | medium | no |  | |  |
| crn | DRSC18755 | medium | no |  | |  |
| deltaCOP | DRSC18760 | medium | no |  | |  |
| gammaCOP | DRSC16955 | medium | no |  | |  |
| geminin | DRSC04984 | medium | no |  | |  |
| His4r | DRSC16703 | medium | no |  | |  |
| hoip | DRSC03546 | medium | no |  | |  |
| ncd | DRSC17012 | medium | no |  | |  |
| Pabp2 | DRSC07501 | medium | no |  | |  |
| Pka-C2 | DRSC20280 | medium | no |  | |  |
| RpII215 | DRSC06100 | medium | no |  | |  |
| RpL10Ab | DRSC10798 | medium | no |  | |  |
| RpL11 | DRSC07537 | medium | no |  | |  |
| RpL12 | DRSC04344 | medium | no |  | |  |
| RpL18A | DRSC07538 | medium | no |  | |  |
| RpL19 | DRSC04649 | medium | no |  | |  |
| RpL22 | DRSC18707 | medium | no |  | |  |
| RpL27A | DRSC00781 | medium | no |  | |  |
| RpL3 | DRSC16834 | medium | no |  | |  |
| RpL31 | DRSC06716 | medium | no |  | |  |
| RpL32 | DRSC16835 | medium | no |  | |  |
| RpL35 | DRSC18347 | medium | no |  | |  |
| RpL7 | DRSC03417 | medium | no |  | |  |
| RpL9 | DRSC03418 | medium | no |  | |  |
| Rpn6 | DRSC07541 | medium | no |  | |  |
| RpS13 | DRSC03419 | medium | no |  | |  |
| RpS14b | DRSC18711 | medium | no |  | |  |
| RpS15Ab | DRSC06129 | medium | no |  | |  |
| RpS19a | DRSC20281 | medium | no |  | |  |
| RpS26 | DRSC03420 | medium | no |  | |  |
| RpS30 | DRSC15119 | medium | no |  | |  |
| RpS4 | DRSC11272 | medium | no |  | |  |
| RpS6 | DRSC18712 | medium | no |  | |  |
| RpS7 | DRSC15394 | medium | no |  | |  |
| RpS8 | DRSC16318 | medium | no |  | |  |
| shi | DRSC20373 | medium | yes | Dynamin (Thompson et al. 2002) | | (Kiger et al. 2003) |
| tsr | DRSC04718 | medium | yes | Cofilin (Gunsalus et al. 1995; Oegema et al. 2000) | | (Somma et al. 2002; Kiger et al. 2003; Rogers et al. 2003) |
| Wnt4 | DRSC00976 | medium | no |  | |  |
| zetaCOP | DRSC11412 | medium | no |  | |  |
|  | DRSC07932 | medium | no |  | |  |
| Act87E | DRSC14104 | weak | yes | Actin (Schroeder 1973) | |  |
| Chc | DRSC20229 | weak | yes | Clathrin heavy chain (Niswonger and O'Halloran 1997) | |  |
| fwd | DRSC08580 | weak | yes | 1-phosphatidylinositol 4-kinase  (Brill et al. 2000) | |  |
| rok | DRSC20277 | weak | yes | Rho kinase (Kosako et al. 2000) | |  |
| sqh | DRSC18837 | weak | yes | Myosin II regulatory light chain (Karess et al. 1991) | | (Somma et al. 2002) |
| Syx5 | DRSC03432 | weak | yes | Syntaxin5 (Xu et al. 2002) | |  |

## References

Adams RR, Tavares AA, Salzberg A, Bellen HJ, Glover DM (1998) pavarotti encodes a kinesin-like protein required to organize the central spindle and contractile ring for cytokinesis. Genes Dev 12(10): 1483-1494.

Brill JA, Hime GR, Scharer-Schuksz M, Fuller MT (2000) A phospholipid kinase regulates actin organization and intercellular bridge formation during germline cytokinesis. Development 127(17): 3855-3864.

Castrillon DH, Wasserman SA (1994) Diaphanous is required for cytokinesis in Drosophila and shares domains of similarity with the products of the limb deformity gene. Development 120(12): 3367-3377.

De Lozanne A, Spudich JA (1987) Disruption of the Dictyostelium myosin heavy chain gene by homologous recombination. Science 236(4805): 1086-1091.

Goshima G, Vale RD (2003) The roles of microtubule-based motor proteins in mitosis: comprehensive RNAi analysis in the Drosophila S2 cell line. J Cell Biol 162(6): 1003-1016.

Gunsalus KC, Bonaccorsi S, Williams E, Verni F, Gatti M, et al. (1995) Mutations in twinstar, a Drosophila gene encoding a cofilin/ADF homologue, result in defects in centrosome migration and cytokinesis. J Cell Biol 131(5): 1243-1259.

Hirose K, Kawashima T, Iwamoto I, Nosaka T, Kitamura T (2001) MgcRacGAP is involved in cytokinesis through associating with mitotic spindle and midbody. J Biol Chem 276(8): 5821-5828.

Karess RE, Chang XJ, Edwards KA, Kulkarni S, Aguilera I, et al. (1991) The regulatory light chain of nonmuscle myosin is encoded by spaghetti-squash, a gene required for cytokinesis in Drosophila. Cell 65(7): 1177-1189.

Kiger A, Baum B, Jones S, Jones M, Coulson A, et al. (2003) A functional genomic analysis of cell morphology using RNA interference. J Biol 2(4): 27.

Knecht DA, Loomis WF (1987) Antisense RNA inactivation of myosin heavy chain gene expression in Dictyostelium discoideum. Science 236(4805): 1081-1086.

Kosako H, Yoshida T, Matsumura F, Ishizaki T, Narumiya S, et al. (2000) Rho-kinase/ROCK is involved in cytokinesis through the phosphorylation of myosin light chain and not ezrin/radixin/moesin proteins at the cleavage furrow. Oncogene 19(52): 6059-6064.

Mabuchi I, Okuno M (1977) The effect of myosin antibody on the division of starfish blastomeres. J Cell Biol 74(1): 251-263.

Mabuchi I, Hamaguchi Y, Fujimoto H, Morii N, Mishima M, et al. (1993) A rho-like protein is involved in the organisation of the contractile ring in dividing sand dollar eggs. Zygote 1(4): 325-331.

Madaule P, Eda M, Watanabe N, Fujisawa K, Matsuoka T, et al. (1998) Role of citron kinase as a target of the small GTPase Rho in cytokinesis. Nature 394(6692): 491-494.

Nislow C, Lombillo VA, Kuriyama R, McIntosh JR (1992) A plus-end-directed motor enzyme that moves antiparallel microtubules in vitro localizes to the interzone of mitotic spindles. Nature 359(6395): 543-547.

Niswonger ML, O'Halloran TJ (1997) A novel role for clathrin in cytokinesis. Proc Natl Acad Sci U S A 94(16): 8575-8578.

Oegema K, Savoian MS, Mitchison TJ, Field CM (2000) Functional analysis of a human homologue of the Drosophila actin binding protein anillin suggests a role in cytokinesis. J Cell Biol 150(3): 539-552.

Prokopenko SN, Brumby A, O'Keefe L, Prior L, He Y, et al. (1999) A putative exchange factor for Rho1 GTPase is required for initiation of cytokinesis in Drosophila. Genes Dev 13(17): 2301-2314.

Rogers SL, Wiedemann U, Stuurman N, Vale RD (2003) Molecular requirements for actin-based lamella formation in Drosophila S2 cells. J Cell Biol 162(6): 1079-1088.

Schroeder TE (1973) Actin in dividing cells: contractile ring filaments bind heavy meromyosin. Proc Natl Acad Sci U S A 70(6): 1688-1692.

Somers WG, Saint R (2003) A RhoGEF and Rho family GTPase-activating protein complex links the contractile ring to cortical microtubules at the onset of cytokinesis. Dev Cell 4(1): 29-39.

Somma MP, Fasulo B, Cenci G, Cundari E, Gatti M (2002) Molecular dissection of cytokinesis by RNA interference in Drosophila cultured cells. Mol Biol Cell 13(7): 2448-2460.

Terada Y, Tatsuka M, Suzuki F, Yasuda Y, Fujita S, et al. (1998) AIM-1: a mammalian midbody-associated protein required for cytokinesis. Embo J 17(3): 667-676.

Thompson HM, Skop AR, Euteneuer U, Meyer BJ, McNiven MA (2002) The large GTPase dynamin associates with the spindle midzone and is required for cytokinesis. Curr Biol 12(24): 2111-2117.

Verni F, Somma MP, Gunsalus KC, Bonaccorsi S, Belloni G, et al. (2004) Feo, the Drosophila Homolog of PRC1, Is Required for Central-Spindle Formation and Cytokinesis. Curr Biol 14(17): 1569-1575.

Watanabe N, Madaule P, Reid T, Ishizaki T, Watanabe G, et al. (1997) p140mDia, a mammalian homolog of Drosophila diaphanous, is a target protein for Rho small GTPase and is a ligand for profilin. Embo J 16(11): 3044-3056.

Xu H, Brill JA, Hsien J, McBride R, Boulianne GL, et al. (2002) Syntaxin 5 is required for cytokinesis and spermatid differentiation in Drosophila. Dev Biol 251(2): 294-306.
