# Supplementary material for: Parallel Chemical Genetic and Genome-Wide RNAi Screens Identify Cytokinesis Inhibitors and Targets
Source: PLoS Biol. 2004 Oct 5;2(12):e379. doi: 10.1371/journal.pbio.0020379 (PMC528723; doi:10.1371/journal.pbio.0020379)
Supplement: Supplementary file 7 [file pbio.0020379.sg002.doc]

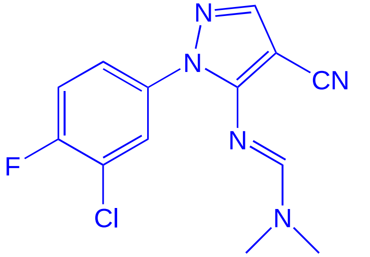

active at 20 μM

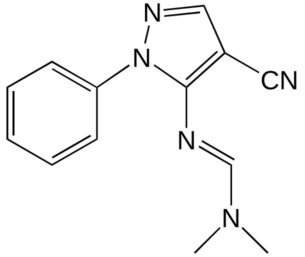

αχτιϖε ατ 100 μM

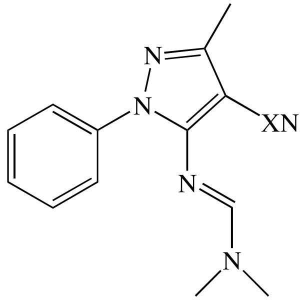

νο εφφεχτ ατ 500 μM

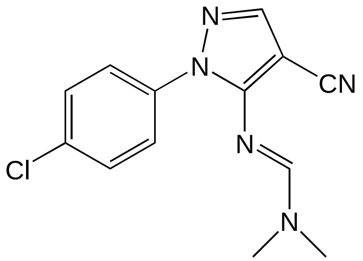

μ οστλψ δεαδ χελλσ, σομε βινυχλεατεσ ατ 500 μM

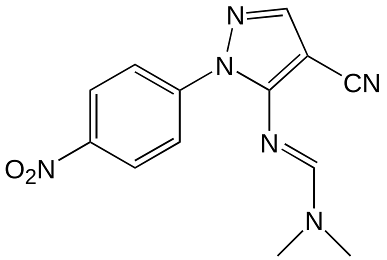

νο εφφεχτ ατ 500 μM

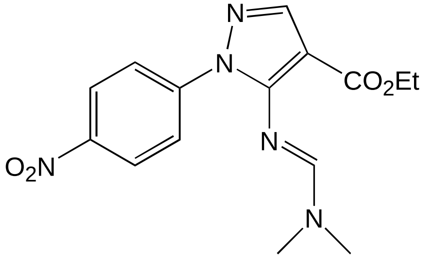

μ οστλψ δεαδ χελλσ, σομε βινυχλεατεσ ατ 500 μM

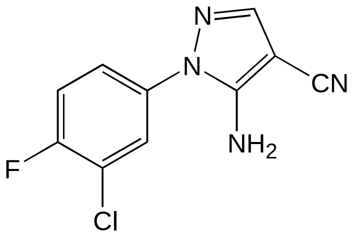

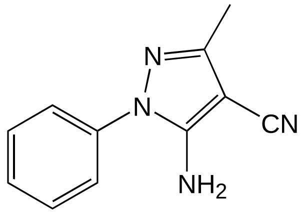

μ οστλψ δεαδ χελλσ, σομε βινυχλεατεσ ατ 500 μM
