# Supplementary material for: Parallel Chemical Genetic and Genome-Wide RNAi Screens Identify Cytokinesis Inhibitors and Targets
Source: PLoS Biol. 2004 Oct 5;2(12):e379. doi: 10.1371/journal.pbio.0020379 (PMC528723; doi:10.1371/journal.pbio.0020379)
Supplement: Supplementary file 8 [file pbio.0020379.sg003.doc]

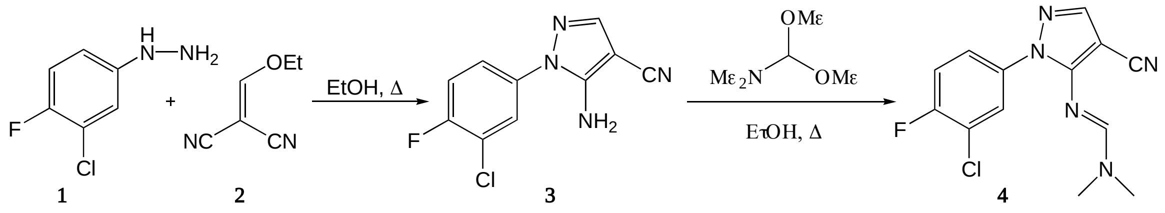

EtOH, Δ
OMe
Me2N
OMe
EtOH, Δ
1
2
3
4
